# On the utility of cerebrospinal fluid biomarkers in canine neurological disorders

**DOI:** 10.1038/s41598-024-73812-y

**Published:** 2024-10-15

**Authors:** Tomas Smolek, Zuzana Vince-Kazmerova, Jozef Hanes, Eva Stevens, Viktor Palus, Ivo Hajek, Stanislav Katina, Petr Novak, Norbert Zilka

**Affiliations:** 1 Institute of Neuroimmunology, Slovak Academy of Sciences, Dúbravská Cesta 9, Bratislava, Slovak Republic; 2 Neuroimmunology Institute, n.p.o., Dvořákovo nábrežie 7527/10, 811 02 Bratislava, Slovak Republic; 3 Axon Neuroscience R&D Services SE, Dvořakovo Nabrezie 10, Bratislava, Slovak Republic; 4 Neurovet –Referral Center for Veterinary Neurology, Bratislavska 2196/32, Trencin, Slovak Republic; 5 Small Animal Referral Centre Sibra, Na Vrátkach 13, Bratislava, Slovak Republic; 6 Institute of Mathematics and Statistics, Masaryk University, Kotlářská 267/2, 611 37 Brno, Czech Republic

**Keywords:** Cerebral biomarkers, Meningoencephalitis, Myelopathies, Tumors, Diseases of the nervous system, Animal physiology, Cancer, Inflammation

## Abstract

The cerebral biomarkers, neurofilament light chain (NfL), amyloid-β, tau, and neuron specific enolase (NSE) reflect a wide spectrum of neurological damage in the brain and spinal cord. With this study, we aimed to assess whether these biomarkers hold any potential diagnostic value for the three most common canine neurological diseases. Canines suffering from meningoencephalitis of unknown origin (MUO), brain tumors, and selected non-infectious myelopathies were included. For each diagnosis, we analyzed these biomarkers in the cerebrospinal fluid collected via cranial puncture from the cisterna magna. Elevated levels of CSF tau, NfL, and NSE were observed in MUO, with all three biomarkers being intercorrelated. Tau and NSE were increased while amyloid-β was decreased in dogs suffering from tumors. In contrast, no biomarker changes were observed in dogs with myelopathies. Covariates such as age, sex, or castration had minimal impact. CSF biomarkers may reflect molecular changes related to MUO and tumors, but not to non-infectious myelopathies. The combination of NfL, tau, and NSE may represent useful biomarkers for MUO as they reflect the same pathology and are not influenced by age.

## Introduction

To date, the diagnosis neurological disorders of the central nervous system (CNS) constitutes a considerable challenge for human and veterinary medicine^1–3^. Thorough clinical evaluation and structural imaging are an essential part of diagnostic algorithms for human CNS disorders. Yet, especially in earlier stages of neurodegenerative disorders, these tools are insufficient to diagnose diffuse processes such as neurodegeneration^4^, and do not allow the assessment of the underlying pathophysiological changes that are responsible for the observed atrophy and neurological deficits, with negative impact on both drug research^5,6^ and clinical practice. Identification of fluid biomarkers (either in cerebrospinal fluid or blood) which would be specific for individual neurological diseases and disease related pathologies has significantly contributed to drug development, early detection and monitoring of neurodegenerative conditions like Alzheimer’s Disease^7^, and could improve the diagnostic accuracy of brain disorders in veterinary practice as well, via complementing established diagnostic algorithms that are based on clinical assessment and structural imaging. Given the fact that canine disorders such as meningoencephalitis of unknown origin (MUO) are often fatal if left untreated^8,9^, accurate diagnosis is imperative to allow timely intervention, achieve remission, and increase survival^10^. In some instances, MUO is not readily distinguishable via established methodologies, it is worthwhile to consider other tools that could be incorporated in the arsenal of the veterinary clinician—such as fluid biomarkers. These are widely used for the diagnostics and prognostics of human neurodegenerative diseases such as Alzheimer’s and Parkinson’s disease, or multiple sclerosis^11,12^. Tau and amyloid-β in the cerebrospinal fluid (CSF) represent cornerstone biomarkers for Alzheimer’s disease diagnostics, displaying very high specificity and sensitivity^13–15^, while NfL in the blood reflects disease activity and therapeutic efficacy in multiple sclerosis^16^; ultimately, studies on CSF biomarkers have led to the development of corresponding assays for blood biomarkers^7^, which are substantially less invasive, and more practical as well. In contrast, fluid biomarkers are used only marginally in veterinary practice with the notable exception of spinal cord injury prognostication^17,18^; most studies evaluating their diagnostic utility are focused predominantly on a single biomarker in one specific neurological disorder^19–22^. So far, only a few studies utilized a combined panel of biomarkers to diagnose neurological disorders, with main focus on assay sensitivity, specificity, and cut-offs to improve diagnostic accuracy^23^. Noteworthy exceptions include studies such as work done by Olby et al., to identify predictors of recovery from spinal cord injury^24^.

Based on initial feasibility assessment and review of literature^18,22,25,26^, four CSF biomarkers—tau, amyloid-β, NSE, and NfL—appeared to be the most suitable for a multi-marker study in dogs. In the present study, we tested these markers in the CSF of canines with non-infectious neurological disorders such as MUO, brain tumors, and selected non-infectious myelopathies. We analyzed their levels, and their ability to distinguish between the individual diagnoses.

## Material and methods

### Study design/case selection

CSF samples evaluated during the study were collected between September 2019 and June 2021. Client-owned dogs were recruited consecutively during the study at referral neurology clinics in Slovakia. CSF sampling were performed by experienced veterinary neurologists.

### Diagnosis

Each patient underwent clinical and neurological examination with complete hematology and biochemistry assessment (concentrations of total protein, glucose, urea, creatinine, ALT, and ALP were measured).

Diagnosis of presumptive Meningoencephalitis of Unknown Origin (MUO) was made in accordance to previously described criteria^27^ based on clinical symptoms, neurological deficits and magnetic resonance imaging (MRI); for a case to be defined as MUO at least part of the suspected inflammatory lesion had to be located intracranially. A total of 47 dogs were diagnosed with (MUO), 19 dogs with (mostly intra-axial, N = 15) brain tumors (TUMORS) and 29 dogs with other non-infectious CNS diseases—Myelopathies and conditions leading to CSF flow abnormalities (Chiari malformation—CM, Syringomyelia—SM, Myelitis—M) were pooled for the purpose of this study under one experimental group called myelopathies (CM/SM/M); this should be considered a ‘miscellaneous’ group as there are differences between these three disorders. Cases of Steroid-Responsive Meningitis-Arteritis (SRMA) were excluded from the study. As owners were unwilling to let healthy dogs undergo anesthesia and CSF sampling, 66 dogs with Idiopathic Epilepsy (IE) were used as the control group instead (CTRL). See Table 1 for cohort characteristics.

**Table 1:** Characteristics of the sample and its cohorts.

	MUO (n = 47)	CM/SM/M (n = 29)	Tumors (n = 19)	Epileptic control (n = 66)
Sex—f/m	25/22	13/16	8/11	26/40
Age—median (range)	8.0 (1.0, 15.5)	5.0 (0.5, 12.0)	11.5 (6.5, 12.5)	6.5 (0.5, 16.0)
Aβ1-42 (pg/mL) (Fig. 1A)				
n	45	27	19	65
Mean, SD	1597.35 (564.93)	1820.93 (450.04)	1256.29 (305.80)	1833.79 (435.71)
95% CI	1427.62, 1767.07	1642,90 1998.96	1108.90, 1403.69	1725.82, 1941.75
NfL (pg/mL) Fig. 1B				
n	30	20	18	48
Mean, SD	21,556.00 (25,169.66)	11,247.10 (15,914.85)	5982.15 (3862.96)	1201.02 (994.89)
95% CI	12,157.50, 30,954.51	3798.72, 18,695.48	4061.15, 7903.16	912.14, 1489.91
NSE (pg/mL) Fig. 1C				
n	30	21	18	48
Mean, SD	47.83 (42.84)	46.44 (40.79)	83.23 (48.59)	31.15 (31.33)
95% CI	31.84, 63.83	27.87, 65.00	59.07, 107.39	22.05, 40.25
Total tau (pg/mL) (Fig. 1D)				
n	45	29	19	65
Mean, SD	82.92 (67.19)	48.65 (29.68)	68.60 (36.63)	31.45 (10.43)
95% CI	62.73, 103.10	37.36, 59.94	50.94, 86.25	28.87, 34.04

Aβ1-42 = Amyloid-β_1–42_; NSE, neuron-specific enolase; NfL, neurofilament-light chain; n, number of dogs or analyzed CSF samples; MUO, Meningoencephalitis of unknown origin; Tumors, brain tumors; CM/SM/M, myelopathies (Chiari malformation, CM; Syringomyelia, SM; Myelitis, M); Epileptic Control, Idiopathic Epilepsy, used as a control group.

Where indicated, the geographically most common infectious CNS diseases (Canine Distemper Virus, Tick-borne encephalitis, Anaplasmosis, Ehrlichiosis, Neosporosis, Toxoplasmosis) were excluded via CSF analysis in an external laboratory (Laboklin, Germany). In the cases where this could not be performed due to the financial restraints of the owners, a presumptive MUO diagnosis was made on the base of the satisfactory clinical response to systemic immunomodulatory treatment.

The presumptive diagnosis of tumors was based on MRI in accordance with previously described MR features of various tumors^28,29^. A definitive diagnosis was not possible, as tumour biopsy was deemed to be too invasive, and henceforth was declined by the owners.

Diagnoses of presumptive non-infectious myelopathies (CM/SM/M) were made in accordance with previously described criteria^27,30–32^. Presumptive diagnosis was made on the base of the signalment, breed predisposition, neurological deficits, laboratory workup, MRI findings and results of CSF analysis. In some cases, infectious CNS diseases were excluded via the analysis of CSF in the external laboratory.

Diagnosis of idiopathic epilepsy was made based on a history of two or more unprovoked epileptic seizures occurring at least 24 h apart, age at epileptic seizure onset of between six months and six years, unremarkable inter-ictal physical and neurological examination, no significant abnormalities on blood tests and urinalysis, and unremarkable fasting and post-prandial bile acids, MRI of the brain and CSF analysis^33^.

### Collection of cerebrospinal fluid

Patients were anesthetized at veterinary clinics (premedication with opioids—butorfanol (Butomidor 10mg/ml, Richter Pharma AG, Wels, Austria), induction with propofol (Proposure 10mg/ml, Axience, Pantin, France), maintenance with isoflurane (IsoFlo 100% w/w, Zoetis, Prague, Czech Republic) with endotracheal intubation. CSF was collected via cranial puncture from the cerebellomedullar cistern (cisterna magna) in accordance with previously described technique^34^.

For each CSF sample, protein concentration, total nucleated cell count (TNCC) and differential cell count were assessed. Protein concentration was identified by Pandy´s reaction and TNCC was determined by Fuchs-Rosenthal counting chamber. In cases of CSF blood contamination during collection, a corrected estimate of the TNCC was done by subtracting one total nucleated cell for every 500 red blood cells^34–37^. When possible, differential cell counts were based on 100 cells counted from slides prepared from cytocentrifugation. Abnormal CSF was defined by the presence of pleocytosis or an abnormal CSF differential cell count. The cut-off value for a pleocytosis was TNCC over 5 cells/µL^34,38,39^. Remaining CSF samples were centrifuged at 1600 g for 10 min., frozen in polypropylene tubes (Sarstedt, Germany) at − 20 °C for ~ 2 months, transferred on dry ice, and stored at − 80 °C until analysis.

### Measurement of protein concentration in CSF

The levels of Amyloid Beta-42 (Aβ42), total Tau (t-Tau, HTAU), Neuron-Specific Enolase (NSE) and neurofilament-light chain (NfL) in CSF were measured using commercially available single-parameter ELISA kits: Aβ42 by INNOTEST® β-AMYLOID(1–42)^25^, t-Tau by INNOTEST® hTAU Ag (both Fujirebio, Gent, Belgium)^18^, NSE by Neuron Specific Enolase ELISA (ALPCO, Salem, USA)^26^ and NfL by NF-light® ELISA (Umman Diagnostics, Umea, Sweden)^22^ according to the manufacturer recommendation (see Supplementary Table 1). Measurements were done in duplicates. Post-analysis evaluation was done by GraphPad Prism 8 (GraphPad Software, CA, USA).

### Statistical analysis

Data processing and statistical analyses were performed in the R version 4.2.2 programming environment^40^. All alternative hypotheses were two-sided and statistical tests were performed at a significance level equal to 0.05. All empirical confidence intervals (CI) are Wald-type, 95%, and two-sided. All p-values and CIs are reported without correction for multiplicity, i.e., without adjustment.

Since measurements of all concentrations of four CSF proteins were done in duplicates, these were averaged, and these arithmetic averages were used in the subsequent statistical analyses. If one or both values were above the upper limit, the upper limit value was used., e.g., for Amyloid β1-42 “ > 4238”, Total tau “ > 2198.5”, Neurofilament-light chain “ > 40,000” (no upper limit for Neuron-specific enolase). To handle outliers, the concentrations were winsorized for each CSF protein and diagnosis using the Tukey quartile rule on both sides of the distribution (owing to symmetry), i.e., the values smaller than the first quartile minus 1.5 times of the interquartile range (IQR) and greater than the third quartile plus 1.5 times of the IQR were set at these limits.

The characteristics of the sample were calculated for each diagnosis and epileptic controls for sex (number of females and males), age (in years, median and range), and concentrations of the four CSF proteins (sample size *n*, mean, standard deviation). For CSF proteins, the CIs for expected values are also given. In cases where the lower limit of CI is below zero, it is truncated to zero. The distributions of concentrations of the four CSF proteins were visualized using boxplots.

The effect of interest was the mean difference between all diagnoses and epileptic controls and all pairs of diagnoses. The null hypothesis *mean difference is equal to zero* was tested against the alternative hypothesis *mean difference is not equal to zero* by the two-sample Student *t*-test with Welch degrees of freedom for mean difference^41^. The results are summarized numerically as mean difference, standard deviations of mean difference, *t*-statistics, degrees of freedom, p-value, lower and upper bound of 95% CI for mean difference. The mean differences and the CIs of the mean difference were also visualized graphically.

Furthermore, the mean difference was adjusted by age, castration, and sex using of linear regression models as follows:

1. concentration ~ diagnosis + age,
2. concentration ~ diagnosis + sex,
3. concentration ~ diagnosis + castration.

The results were summarized numerically and graphically as above but using least-squares marginal mean differences, i.e., adjusted mean differences^42^. All regression coefficients and effects, and their variances were estimated by ordinary least squares algorithm.

The linear association between the concentrations of all four CSF proteins was assessed using the Pearson correlation coefficient. The null hypothesis *the correlation coefficient is equal to zero* was tested against the alternative hypothesis *the correlation coefficient is not equal to zero* by Fisher *z*-test^43^. The results were summarized numerically by Pearson correlation coefficient, lower and upper bound of 95% CI for a correlation coefficient calculated using Fisher *z*-transformation, *z*-statistics, and p-value.

To assess the diagnostic ability of all four CSF proteins to distinguish the diagnoses or diagnoses from epileptic controls, the receiver operating characteristic (ROC) analysis was used^44^. The results were summarized numerically by area under the curve (AUC), lower and upper bound of 95% CI for an AUC, cut-off point, optimal sensitivity and specificity with respective lower and upper bound of 95% CI calculated using *logit* transformation. If the optimal sensitivity or specificity is equal to zero or one, related 95% CI is not reported. The ROCs were also visualized graphically.

## Results

### Study sample and cohorts

**Fig. 1:**
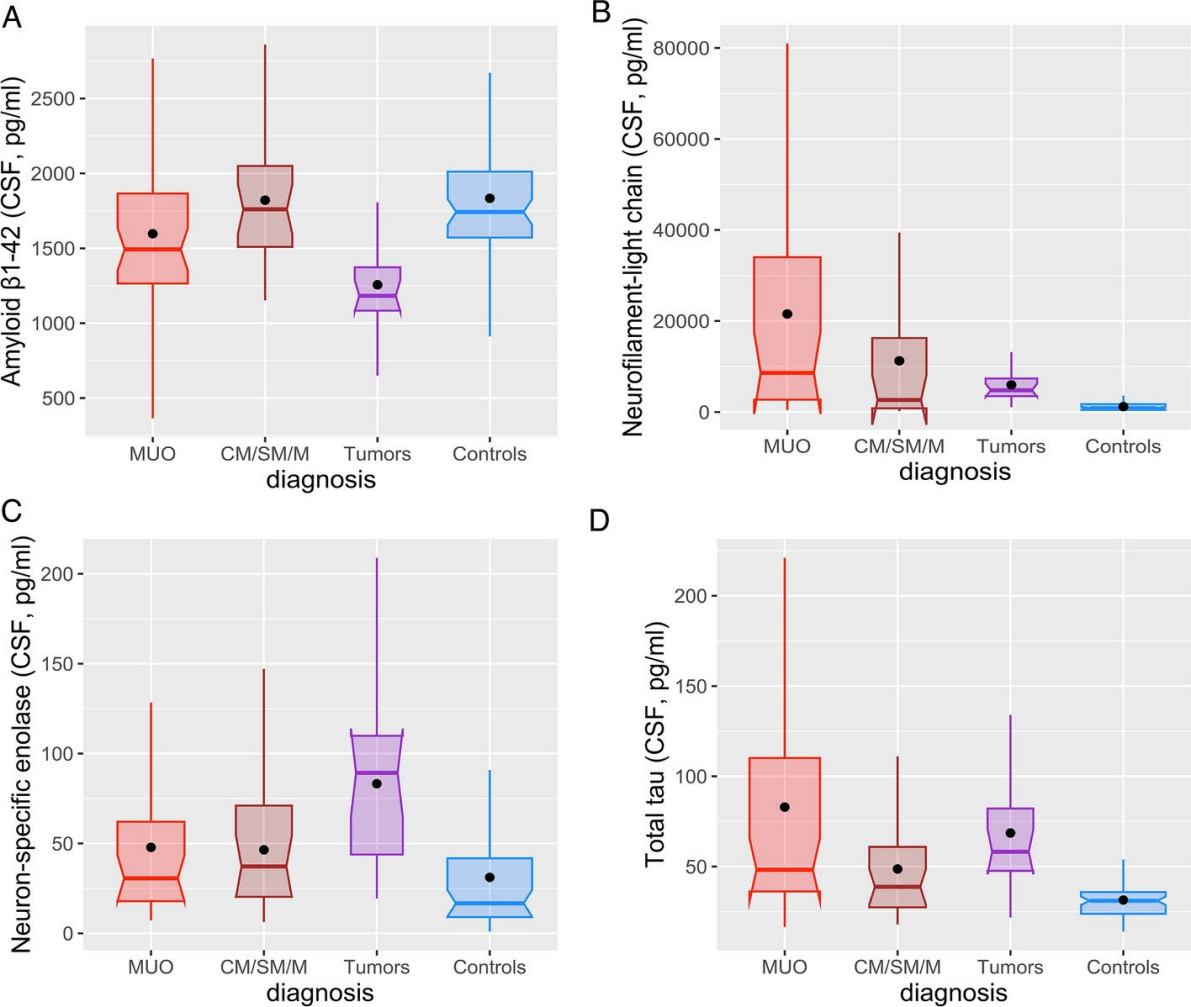
Concentrations of biomarkers in the cerebrospinal fluid of MUO, CM/SM/M, tumor diagnosis and epileptic control group. Aβ1-42 (**A**), NfL (**B**), NSE (**C**), total tau (**D**). Box plots denote median, quartiles, with whiskers showing quartiles ± 1.5 × IQR. Dots denote arithmetic mean.

A total of 161 dogs (72 females, 89 males, median age 7.0 years, age range 0.5–16.0 years) were evaluated. General characteristics of the study cohorts, and descriptive statistics of biomarker levels are presented in Table 1. An illustration is provided in Fig. 1.

An overview of breeds is provided in Supplement 1.

### The effect of covariates on The CSF biomarkers in canine neurological diseases

**Fig. 2:**
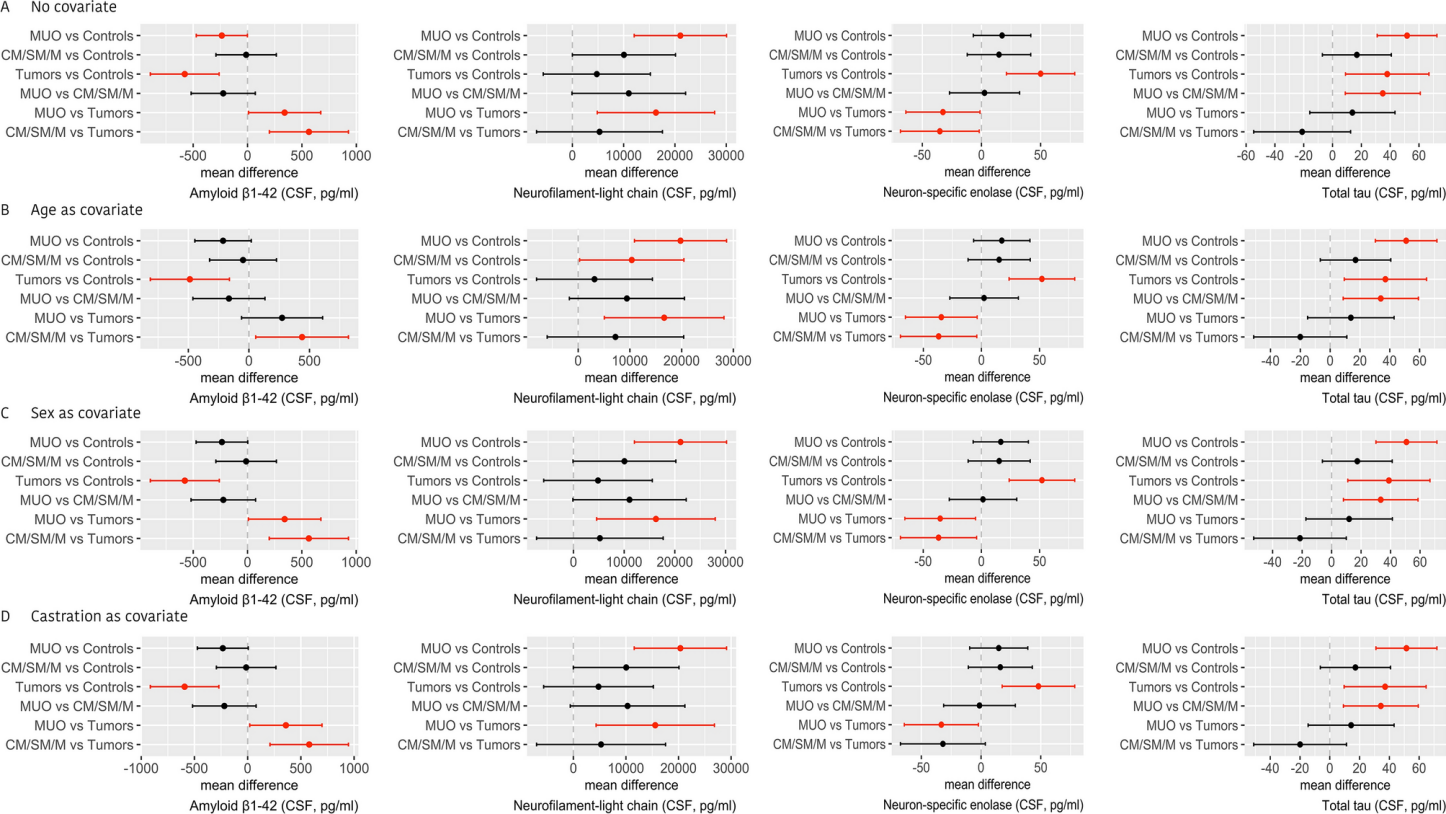
Comparisons of CSF biomarkers between diagnostic groups, with and without covariate adjustment. (**A**) No adjustment, (**B**) adjustment for age, (**C**) adjustment for sex, (**D**) adjustment for castration. See Supplement for details. Forrest plots show 95% CI for differences; CIs that do not include 0 denote significant differences, and are highlighted in red.

The mean **Aβ1-42** concentration was significantly different between the epileptic control group and MUO (mean difference − 236.44, 95% CI − 471.66, − 1.21; SD difference 90.55; *p* = 0.04831), or tumors (mean difference − 577.49, 95% CI − 893.84, − 261.15; SD difference 121.78; *p* = 0.00003). There was no significant difference of **Aβ1-42** concentration in CSF of the CM/SM/M group compared to epileptic controls (mean difference − 12.86, 95% CI − 290.58, − 264.87; SD difference 106.91; *p* = 0.99938). The tumors group also displayed significantly decreased CSF Aβ1-42 compared to the MUO (mean difference 341.05, 95% CI 9.19, 672.92; SD difference 127.75; *p* = 0.04147) and CM/SM/M (mean difference 564.64, 95% CI 201.41, 927.86; SD difference 139.83; *p* = 0.00049) group (Fig. 2A). Adjustment for age reduced the difference between MUO and epileptic controls group (mean difference − 213.35, 95% CI − 446.34, 19.65; SD difference 89.69; *p* = 0.09) and MUO and tumors group to non-significant levels (mean difference 273.97, 95% CI − 60.83, 608.77; SD difference 128.88; *p* = 0.150) (Fig. 2B). Adjustment for sex reduced the difference between MUO and epileptic controls group to non-significant levels (mean difference − 233.27, 95% CI − 472.26, 5.72; SD difference 91.98; *p* = 0.06) ((Fig. 2C).and adjustment for castration reduced the difference between MUO and epileptic controls group to non-significant levels (mean difference − 235.58, 95% CI − 473.39, 2.31; SD difference 91.56; *p* = 0.053) (Fig. 2D).

There was a significant increase of **NfL** in MUO (mean difference 20,354.98, 95% CI 11,564.60, 29,144.36; SD difference 3370.13; *p* ˂ 0.0001) when compared to epileptic controls. In contrast, the **NfL** concentration in CSF did not show significant differences between the epileptic control group and CM/SM/M (mean difference 10,046.08, 95% CI − 4.97, 20,097.12; SD difference 3853.89; *p* = 0.05016) or tumors group (mean difference 4781.13, 95% CI − 5656.63, 15,218.90; SD difference 4002.17; *p* = 0.63151). Except for the MUO vs. tumors comparison (mean difference 15,573.85, 95% CI 4314.42, 26,833.27; SD difference 4317.22; *p* = 0.00258), we did not observe any difference in mean NfL concentration between diagnoses (Fig. 2B). Adjustment for age increased the difference between CM/SM/M and epileptic control group to slight significant levels (mean difference 10,365.82, 95% CI 285.62, 20,446.03; SD difference 3864.55; p = 0.04137) (Fig. 2B). No effects of sex and castration were observed (Fig. 2C and D).

There was a significant increase in **NSE** concentration in Tumors (mean difference 52.08, 95% CI 23.90, 80.27; SD difference 10.81; *p* = 0.00003) when compared with the epileptic control group. No difference was found between MUO vs. epileptic controls (mean difference 16.69, 95% CI − 7.05, 40.42; SD difference 9.10; *p* = 0.26320) or CM/SM/M vs. epileptic controls (mean difference 15.29, 95% CI − 11.39, 41.97; SD difference 10.23; *p* = 0.44418). We found a significant increase in **NSE** in the tumors group when compared to MUO (mean difference − 35.40, 95% CI − 65.80, − 5.00; SD difference 11.66; *p* = 0.01552) or CM/SM/M (mean difference − 39.80, 95% CI − 69.55, − 4.04; SD difference 12.56; *p* = 0.02110). Adjustment for age reduced the difference between CM/SM/M and tumors group to non-significant levels (mean difference − 31.93, 95% CI − 67.45, 3.59; SD difference 13.62; *p* = 0.09429) (Fig. 2B). Effect of sex and castration were not observed (Fig. 2C and D).

We observed a significant increase of **total tau** in MUO (mean difference 51.47, 95% CI 30.99, 71.94; SD difference 7.88; *p* ˂ 0.0001) and tumors (mean difference 37.15, 95% CI 9.61, 64.68; SD difference 10.60; *p* = 0.00333) when compared to the epileptic control group. No significant difference was found between CM/SM/M and epileptic controls (mean difference 17.20, 95% CI − 6.38, 40.77; SD difference 9.08; *p* = 0.23454). We found a significant increase in **total tau** in the MUO when compared to CM/SM/M (mean difference 34.27, 95% CI 9.13, 59.41; SD difference 9.68; p = 0.00294). No differences between other diagnoses were observed. Effect of age, sex and castration were not observed (Fig. 2B–D). Detailed statistical results are listed in the supplement.

### Correlation analyses of CSF biomarkers within diagnoses

In MUO and tumors there was a strong positive correlation between all biomarkers of brain injury (tau, NfL and NSE), but not with amyloid β. In the CM/SM/M group, only total tau and NfL were correlated (see Table 2).

**Table 2:** Correlation analysis between individual biomarkers within each diagnosis.

Diagnosis	Pearson’s r	Amyloid β1-42	Total tau	NfL	NSE
MUO	Amyloid β1-42		0.025	0.072	− 0.019
	Total tau	0.025		**0.606**	**0.742**
	NfL	0.072	**0.606**		**0.519**
	NSE	− 0.019	**0.742**	**0.519**	
CM/SM/M	Amyloid β1-42		− 0.133	− 0.183	0.194
	Total tau	− 0.133		**0.855**	0.088
	NfL	− 0.183	**0.855**		0.055
	NSE	0.194	0.088	0.055	
Tumors	Amyloid β1-42		− 0.324	− 0.158	− 0.287
	Total tau	− 0.324		**0.660**	**0.488**
	NfL	− 0.158	**0.660**		**0.583**
	NSE	− 0.287	**0.488**	**0.583**	
Epileptic control	Amyloid β1-42		0.070	**− 0.324**	0.262
	Total tau	0.070		0.209	0.063
	NfL	**− 0.324**	0.209		**0.371**
	NSE	0.262	0.063	**0.371**	

Pearson’s r—correlation coefficient. Values significant at *p* < 0.05 are displayed in bold. See supplement for detailed correlation statistics.

### Specificity and sensitivity of individual biomarkers in canine neurological disorders

#### MUO vs epileptic controls

**Fig. 3:**
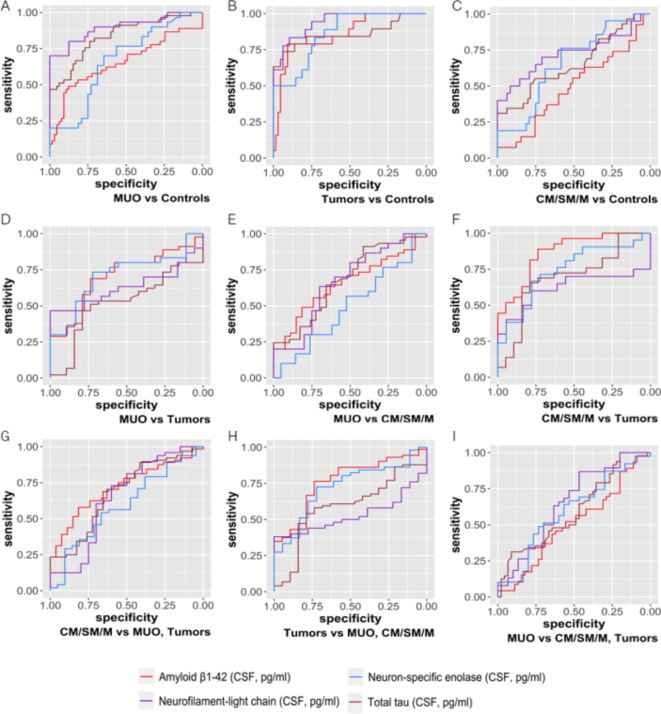
Sensitivity and specificity of Aβ1-42, NfL, NSE and total tau biomarkers for the differentiation between diagnostic groups. Sensitivity and specificity of biomarkers to distinguish between the control group with Idiopathic Epilepsy (controls) and individual diagnoses (MUO, tumors, CM/SM/M) (**A**–**C**), among diagnoses (**D**–**F**), and individual diagnoses vs. the other two (**G**–**I**) is shown. MUO = Meningoencephalitis of Unknown Origin; CM/SM/M, Chiari malformation = CM, Syringomyelia = SM, Myelitis = M; sensitivity = optimal sensitivity (TPF); specificity = optimal specificity (1 − FPF).

**Total tau** discriminated MUO from epileptic controls (AUC 0.84 [95% CI 0.74, 0.90], cut-off 35.67 pg/mL) 80% optimal sensitivity (95% CI 0.65, 0.90) and 74% optimal specificity (95% CI 0.27, 0.96); **NfL** (AUC 0.90 [95% CI 0.79, 0.95], cut-off 3625.4 pg/mL) with 70% optimal sensitivity (95% CI 0.52, 0.84) and 100% optimal specificity; **NSE** (AUC 0.67 [95% CI 0.53, 0.78], cut-off 21.52 pg/mL), with 70% optimal sensitivity (95% CI 0.48, 0.86) and 65% optimal specificity (95% CI 0.38, 0.84); **Aβ1-42** (AUC 0.65 [95% CI 0.54, 0.75], cut-off 1471.28 pg/mL), with 49% optimal sensitivity (95% CI 0.33, 0.65) and 88% optimal specificity (95% CI 0.61, 0.97) (Fig. 3A).

#### Tumors vs epileptic controls

**Aβ1-42** discriminated tumors from epileptic controls (AUC 0.86 [95% CI 0.71, 0.94], cut-off 1444.75 pg/mL), with 79% optimal sensitivity (95% CI 0.55, 0.92) and 91% optimal specificity (95% CI 0.26, 1); **total tau** protein (AUC 0.86 [95% CI 0.71, 0.94], cut-off 48.45 pg/mL), with 74% optimal sensitivity [95% CI 0.49, 0.89] and 94% optimal specificity [95% CI 0.32, 1]); **NSE** (AUC 0.86 [95% CI 0.71, 0.94], cut-off 19.15 pg/mL), with 100% optimal sensitivity and 58% optimal specificity [95% CI 0.44, 0.71]); **NfL** (AUC 0.94 [95% CI 0.8, 0.98], cut-off 2837.45 pg/mL), with 83% optimal sensitivity (95% CI 0.55, 0.95) and 90% optimal specificity (95% CI 0.53, 0.99) (Fig. 3B).

#### CM/SM/M vs epileptic controls

**NfL** discriminated CM/SM/M from epileptic controls (AUC 0.73 [95% CI 0.57, 0.84], cut-off 2911.43 pg/mL), with 50% optimal sensitivity (95% CI 0.29, 0.71) and 92% optimal specificity (95% CI 0.02, 1); **Aβ1-42** (AUC 0.49 [95% CI 0.36, 0.62], cut-off 1486.58 pg/mL), with 74% optimal sensitivity (95% CI 0.53, 0.88) and 14% optimal specificity (95% CI 0.03, 0.47); **total tau** protein (AUC 0.66 [95% CI 0.53, 0.77], cut-off 54.16 pg/mL), with 31% optimal sensitivity (95% CI 0.17, 0.50) and 100% optimal specificity; **NSE** (AUC 0.66 [95% CI 0.50, 0.79], cut-off 19.62 pg/mL), with 76% optimal sensitivity (95% CI 0.51, 0.91) and optimal specificity 58% (95% CI 0.27, 0.84) (Fig. 3C).

#### MUO vs tumors

**Aβ1-42** discriminated MUO from tumors (AUC 0.71 [95% CI 0.56, 0.82], cut-off 1315.65 pg/mL) 69% optimal sensitivity (95% CI 0.47, 0.85) and 74% optimal specificity (95% CI 0.41, 0.92); **NfL** (AUC 0.63 [95% CI 0.46, 0.77], cut-off 14,134.72 pg/mL) with 47% optimal sensitivity (95% CI 0.30, 0.64) and 100% optimal specificity; **NSE** (AUC 0.71 [95% CI 0.55, 0.84], cut-off 51.02 pg/mL), with 73% optimal sensitivity (95% CI 0.40, 0.92) and 72% optimal specificity (95% CI 0.43, 0.90); **total tau** protein(AUC 0.52 [95% CI 0.37, 0.67], cut-off 46.38 pg/mL), with 47% optimal sensitivity (95% CI 0.28, 0.67) and 79% optimal specificity (95% CI 0.44, 0.95) (Fig. 3D).

#### MUO vs CM/SM/M

**Aβ1-42** discriminated MUO from CM/SM/M (AUC 0.65 [95% CI 0.52, 0.77], cut-off 1471.95 pg/mL) 49% optimal sensitivity (95% CI 0.29, 0.70) and 82% optimal specificity (95% CI 0.54, 0.94); **NfL** (AUC 0.67 [95% CI 0.50, 0.80], cut-off 4821.3 pg/mL) with 63% optimal sensitivity (95% CI 0.42, 0.80) and 70% optimal specificity (95% CI 0.26, 0.94); **NSE** (AUC 0.50 [95% CI 0.34, 0.65], cut-off 103.32 pg/mL), with 80% optimal sensitivity (95% CI 0.58, 0.92) and 10% optimal specificity (95% CI 0.01, 0.59); **total tau protein** (AUC 0.68 [95% CI 0.55, 0.79], cut-off 31.80 pg/mL), with 91% optimal sensitivity (95% CI 0.70, 0.98) and 41% optimal specificity (95% CI 0.20, 0.66) (Fig. 3E).

#### CM/SM/M vs tumors

**Aβ1-42** discriminated CM/SM/M from tumors (AUC 0.87 [95% CI 0.73, 0.94], cut-off 1309.08 pg/mL) 89% optimal sensitivity (95% CI 0.63, 0.97) and 74% optimal specificity (95% CI 0.36, 0.93); **NfL** (AUC 0.62 [95% CI 0.43, 0.78], cut-off 3379.07 pg/mL) with 60% optimal sensitivity (95% CI 0.37, 0.79) and 78% optimal specificity (95% CI 0.10, 0.99); **NSE** (AUC 0.74 [95% CI 0.56, 0.87], cut-off 40.00 pg/mL), with 67% optimal sensitivity (95% CI 0.26, 0.92) and 78% optimal specificity (95% CI 0.49, 0.93); **total tau** protein(AUC 0.69 [95% CI 0.53, 0.82], cut-off 44.40 pg/mL), with 66% optimal sensitivity (95% CI 0.37, 0.86) and 79% optimal specificity (95% CI 0.47, 0.94) (Fig. 3F).

#### CM/SM/M vs MUO, tumors

**Aβ1-42** discriminated CM/SM/M for other two clinical diagnosis (AUC 0.72 [95% CI 0.58, 0.82], cut-off 1471.95 pg/mL, with 82% optimal sensitivity [95% CI 0.56, 0.94], and 58% optimal specificity [95% CI 0.38, 0.75]); **total tau** protein (AUC 0.68 [95% CI 0.55, 0.79], cut-off 42.47 pg/mL, with 66% optimal sensitivity [95% CI 0.42, 0.84], and 69% optimal specificity [95% CI 0.49, 0.84]); **NfL** (AUC 0.65 [95% CI 0.49, 0.78], cut-off 3379.07 pg/mL, with 60% optimal sensitivity [95% CI 0.35, 0.81], and 73% optimal specificity [95% CI 0.42, 0.91]); **NSE** (AUC 0.59 [95% CI 0.44, 0.73], cut-off 40.00 pg/mL, with 67% optimal sensitivity [95% CI 0.38, 0.87], 54% optimal specificity [95% CI 0.33, 0.74]) (Fig. 3G).

#### Tumors vs MUO, CM/SM/M

**Aβ1-42** discriminated tumors from other clinical diagnoses (AUC 0.77 [95% CI 0.61, 0.87], cut-off 1309.08 pg/mL, with 74% optimal sensitivity [95% CI 0.43, 0.91], and 76% optimal specificity [95% CI 0.58, 0.89]); **NSE** (AUC 0.73 [95% CI 0.56, 0.85], cut-off 51.02 pg/mL, with 72% optimal sensitivity [95% CI 0.45, 0.89], and 73% optimal specificity [95% CI 0.41, 0.91]); **total tau** protein (AUC 0.59 [95% CI 0.44, 0.73], cut-off 46.38 pg/mL, with 79% optimal sensitivity [95% CI 0.51, 0.93], and 54% optimal specificity [95% CI 0.33, 0.74]); **NfL** (AUC 0.53 [95% CI 0.38, 0.68], cut-off 14,134.72 pg/mL, with 100% optimal sensitivity and 38% optimal specificity [95% CI 0.26, 0.52]) (Fig. 3H).

#### MUO vs CM/SM/M, tumors

**Aβ1-42** discriminated MUO from other two clinical diagnosis (AUC 0.51 [95% CI 0.39, 0.62], cut-off 1198.47 pg/mL, with 20% optimal sensitivity [95% CI 0.08, 0.42] and 72% optimal specificity [95% CI 0.51, 0.86]); **NfL** (AUC 0.65 [95% CI 0.51, 0.77], cut-off 14,134.72 pg/mL, with 47% optimal sensitivity [95% CI 0.26, 0.68] and 87% optimal specificity [95% CI 0.58, 0.97]); **NSE** (AUC 0.60 [95% CI 0.46, 0.72], cut-off 51.02 pg/mL, with 73% optimal sensitivity [95% CI 0.47, 0.90] and 49% optimal specificity [95% CI 0.28, 0.70]), **total tau** protein (AUC 0.60 [95% CI 0.48, 0.71], cut-off 31.8 pg/mL, with 91% optimal sensitivity [95% CI 0.70, 0.98], 31% optimal specificity [95% CI 0.16, 0.52] (Fig. 3I).

## Discussion

In the present study, we evaluated four CSF biomarkers which are commonly used for diagnostic purposes in human neurologic disorders, and assessed their diagnostic properties in three clinically relevant canine CNS disorders. All selected biomarkers are homologous to their human counterparts^3,45–47^. Tau is an indicator of axonal and neuronal damage, with CSF levels of total tau being positively correlated with the amount of tissue damage in a wide range of CNS conditions, including neurodegenerative disorders, injury, and prionoses^48^. NSE is a highly specific marker for neurons and peripheral neuroendocrine cells^49^. Its levels correlate with neurological and functional severity of traumatic brain and spinal cord injury, brain infarctions, and neuroendocrine tumors in human patients^49–52^ and inflammatory diseases such as GM1 gangliosidosis, encephalitis and canine distemper in dogs^53–55^. NfL is increased in CSF proportionally to the degree of axonal damage in a variety of neurological disorders, including inflammatory, neurodegenerative, traumatic, and cerebrovascular diseases^56^. Amyloid-β is considered to be a biomarker specific for senile amyloid plaques in Alzheimer’s disease; in brains of aged dogs, a large number of amyloid deposits are found^46,57–59^, accompanied by a decrease of amyloid-β in CSF^57,60,61^. Beside amyloid-β, these biomarkers are not specific for individual underlying pathologies, though, with changes occurring in response to a range of CNS disorders and injuries.

We have focused on the most common neurological disorders, meningoencephalitis of unknown origin or etiology (MUO, MUE), brain tumors, and selected non-infectious myelopathies.

Intracranial **tumors** of dogs are frequently encountered in veterinary medicine^62–64^, with incidence reported as 14.5 cases per 100,000 and prevalence being higher in middle-aged to older dogs^65–67^. Current diagnostic algorithms of brain neoplasms utilize clinical examination, imaging methods (CT, MRI) and analysis of selected CSF markers^67–69^.

Interestingly, in our study, we showed a significant decrease of Aβ42 levels in the tumor group and MUO when compared to epileptic controls and other diagnoses. Aβ42 discriminated tumors from epileptic controls with 91% specificity and 79% sensitivity and MUO from epileptic controls with 88% specificity and 49% sensitivity. Age being a crucial factor in the concentration of Aβ42 in CSF, with levels initially increasing with age, then decreasing in an age-dependent fashion^57,60,61^, we have evaluated whether adjusting for age would explain this difference in CSF Aβ42. Despite the age difference between the MUO, tumor group and epileptic controls or other diagnoses, respectively, correcting for age did not have pronounced impact on the results. This suggests a direct link between tumors, MUO and the amyloid-β pathway. Histopathological studies in similarly aged cohorts may shed light on the molecular mechanism; with further optimization of methodology, such studies may support the inclusion of amyloid-β in diagnostic algorithms in the future.

Similarly, we saw increased levels of NSE in tumors, with NSE discriminating tumors from other diagnoses with 72% optimal sensitivity and 73% optimal specificity and from epileptic controls with 100% optimal sensitivity and 58% optimal specificity. This is not sufficient to employ NSE as a single biomarker for diagnostic purposes; including it in a biomarker panel may be feasible though.

**Meningoencephalitis of unknown origin or etiology—**the most frequent canine non-infectious neuroinflammatory brain condition—constitutes 5–25% of all CNS disorders in dogs^70,71^. Affected animals display a variety of neurological signs including seizures. The disorder can presently only be truly confirmed based on histopathology^9,27,72,73^. Diagnosis of probable MUO is based on clinical signs, MRI, CSF analysis and exclusion of infectious agents^27,74^. Given that efficacious treatment options are available, the unmet need for improved diagnostic tools that would allow timely intervention is high.

The present study shows that the investigated CSF biomarkers of neuronal and axonal damage—tau and NfL—were significantly increased in dogs with MUO. On the other hand, Aβ was significantly decreased. Interestingly, there was high inter-correlation between these three biomarkers, suggesting that they may reflect the same pathological changes. However, the diagnostic accuracy of the abovementioned biomarkers was not sufficient to reliably discriminate MUO from other diagnoses. A previous study showed significantly higher levels of NfL in MUO dogs compared to healthy controls, with high sensitivity (> 89%) and specificity (> 96%). However, the healthy cohort in said study was younger than the MUO dogs, and the analysis was not adjusted for age, limiting interpretability^22^.

**Chiari malformation (CM) and Syringomyelia (SM)** are common conditions associated with myelopathies that frequently occur in small, brachycephalic, and toy dogs^75–77^. SM may be present in more than 50% of dogs with CM^78,79^. By definition, CM is a malformation of the skull and cranio-cervical junction which compromises the neural parenchyma to cause pain and/or disrupt CSF circulation, which can result in SM^80^. SM results in the development of fluid-containing cavities within the parenchyma of the spinal cord as a consequence of abnormal cerebrospinal fluid movement^80^. Currently, the diagnoses of CM or SM are based on clinical symptoms and imaging (MRI, CT)^81–83^. **Myelitis** (M) is defined as an inflammation of spinal cord parenchyma or meningomyelitis. Prevalence in veterinary medicine is relatively rare and most often is occurred in combination with inflammatory brain disease^84,85^. A clinical diagnosis of myelitis is typically made by a combination of clinical presentation, MRI, CT imaging of involved part of the CNS and results of cerebrospinal fluid (CSF) analysis^9,84^.

Our data did not show any significant changes of selected biomarkers in myelopathies, with levels of tau, NfL, NSE and Aβ being similar to the epileptic control group. It seems that myelopathies do not cause neuronal damage which could be monitored by these biomarkers. Previously, one study on dogs with intervertebral disc herniation (IVDH) showed significantly higher CSF tau concentrations in dogs showing plegia compared to healthy dogs and dogs with paresis^18^, however the study did not compare dogs with IVDH to other diagnoses.

While previous studies usually employed small cohorts with a single disorder, the present study considerably expands the available evidence base by evaluating a sizable cohort of 161 dogs of a wide range of breeds and assessing multiple biomarkers in multiple disorders. This has allowed assessing the utility of these markers and their combinations in differentiating common canine CNS disorders. Overall, the observed sensitivity, specificity, and ROCs are not yet at the levels of reliable diagnostic assays; further research is necessary before these CSF markers can be incorporated in veterinary diagnostic algorithms alongside clinical and imaging assessments. In their present state, they can be useful for disease monitoring, early pathology detection in neurogenetic conditions etc. Ultimately, it is also desirable to develop blood biomarker assays for dogs, as these are far less invasive and logistically significantly more expedient—and for many biomarkers such as NfL they are highly representative of levels measured in the CSF^86^.

### Limitations

The present study has some limitations:

- Dogs with idiopathic epilepsy were used as a control group instead of healthy individuals, as samples were collected at neurologic referral clinics whose ethical guidelines do not allow anesthesia in healthy individuals without indications that would justify it. The group did not include individuals with refractory epilepsy.
- The study evaluates only diagnoses which are common at Slovak veterinary neurological clinics. A wider spectrum of neurologic disorders would provide greater insight into differences in biomarker patterns between disorders.
- The most relevant differential diagnoses, MUO and Brain Tumors, reach the high probability level only and therefore remain “presumptive/suspected”, as no confirmatory tissue diagnosis could be performed. As such, the ability of biomarkers to discriminate between diagnoses might be hindered by an inaccurate diagnosis.
- The results of centrally sampled biomarkers (e.g., from the cisterna magna) may differ from studies where sampling was performed at lumbar level ^87^, limiting comparability somewhat.

### Conclusions

To sum up, we investigated four biomarkers—tau, Aβ, NfL and NSE—in three canine neurological diseases. The observed diagnostic accuracy was not sufficient to use them as a routine diagnostic or screening tool; however, in the case of MUO a combination of three of them (tau, Aβ and NfL) has potential to improve diagnostic precision. Future studies are warranted to assess their prognostic and theragnostic utility in the treatment of canine MUO.

These biomarkers could also be utilized in studies of molecular pathways involved in various canine CNS diseases.

## Supplementary Information

Supplementary Information.

## Data Availability

Please contact author for data requests.
