# Supplementary material for: On the utility of cerebrospinal fluid biomarkers in canine neurological disorders
Source: Sci Rep. 2024 Oct 15;14:24129. doi: 10.1038/s41598-024-73812-y (PMC11480401; doi:10.1038/s41598-024-73812-y)
Supplement: Supplementary file 1 — Supplementary Information. [file 41598_2024_73812_MOESM1_ESM.docx]

Supplementary material for:

“On the utility of cerebrospinal fluid biomarkers in canine neurological disorders”

Contents

[Details of used ELISA kits 3](#_Toc157783499)

[Detailed statistical analysis results 4](#_Toc157783500)

[Dogs breeds by diagnosis 9](#_Toc157783501)

[Clinical, MRI, and CSF analysis details for individual animals 14](#_Toc157783502)

List of Supplementary Tables

[Supplementary Table 1**.** Evaluated biomarkers and corresponding human ELISA kits used for their measurement in the CSF of dogs 3](#_Toc157783491)

[Supplementary Table 2: CSF biomarkers by Diagnostic Group comparison. **NO Covariate adjustment** 4](#_Toc157783492)

[Supplementary Table 3: CSF biomarkers by Diagnostic Group comparison. **Age Covariate adjustment** 5](#_Toc157783493)

[Supplementary Table 4: CSF biomarkers by Diagnostic Group comparison. **Sex Covariate adjustment** 6](#_Toc157783494)

[Supplementary Table 5: CSF biomarkers by Diagnostic Group comparison. **Castration Covariate adjustment** 7](#_Toc157783495)

[Supplementary Table 6: Intercorrelation of biomarkers by diagnosis (details) 8](#_Toc157783496)

[Supplementary Table 7: Dog breeds by diagnosis 12](#_Toc157783497)

[Supplementary Table 8: Clinical, MRI, and CSF analysis details for individual animals 14](#_Toc157783498)

List of Supplementary Figures

[**Supplementary Figure 1: Multifocal MUO with hyperintense lesions in brain stem and neocortex.** Multiple diffuse T2 hyperintense, T1 isointense lesions with mild saturation postcontrast are present predominantly in the left parietal and occipital lobe and brainstem (red arrows). 9](#_Toc157783737)

[**Supplementary Figure 2: Suspected glioma in the brain stem.** A T2 hyperintense, T1 isointense lesion is observed on the right side of the brainstem. The lesion displays a mass effect and saturation postcontrast. 9](#_Toc157783738)

[**Supplementary Figure 3:** Differential diagnosis: glioma vs. MUO. A right-sided diffuse intra-axial, expansive, T2 hyperintense, T1 isointense lesion in the area of the thalamus is trespassing on the brainstem. The lesion does not display saturation postcontrast in the T1 sequence. 10](#_Toc157783739)

[**Supplementary Figure 4: Syringomyelia with Chiari malformation.** The MRI of the brain and cervical spinal cord shows a caudal malformation of the occipital bone (Chiari-like malformation), herniation and compression of the cerebellum against the brainstem. MRI cervical segments and spinal cord showed also syringomyelia. 10](#_Toc157783740)

[**Supplementary Figure 5: Sagittal T2-weighted MRI scan of the thoraco-lumbar spine and spinal cord.** Arrows indicate a diffuse, T2 and STIR hyperintense, intra-medullar lesion at the T12-T13-L1 level. In T1-weighted images, a massive saturation postcontrast is observed in the meninges. 11](#_Toc157783741)

# Details of used ELISA kits

Supplementary Table 1**.** Evaluated biomarkers and corresponding human ELISA kits used for their measurement in the CSF of dogs

| Biomarker | Kit | Cat.number |
| --- | --- | --- |
| Total Tau (t-TAU) | INNOTEST hTau Ag IVD IVD ELISA kit | 81572 |
| Amyloid Beta-42 (Aβ42) | INNOTEST β-AMYLOID (1–42) IVD ELISA kit | 81576 |
| Neurofilament (NfL) | NF-light ® ELISA (Uman Diagnostics) | UD51001 |
| Neuron specific enolase (NSE) | ALPCO - Neuron Specific Enolase ELISA (NSE ELISA) | 43-NSEHU-E01 |

# Detailed statistical analysis results

Supplementary Table 2: CSF biomarkers by Diagnostic Group comparison. **NO Covariate adjustment**

| **protein** | **contrast** | **mean diff** | **sd diff** | **lower** | **upper** | **t-statistic** | **df** | **p-value** | **significant** |
| --- | --- | --- | --- | --- | --- | --- | --- | --- | --- |
| Amyloid β1-42 (CSF) | MUO vs Controls | -236.438 | 90.5525 | -471.6639 | -1.2113 | -2.611 | 152 | 0.04831 | TRUE |
| Total tau (CSF) | MUO vs Controls | 51.465 | 7.8818 | 30.9941 | 71.9368 | 6.530 | 154 | 0.00000 | TRUE |
| Neurofilament-light chain (CSF) | MUO vs Controls | 20354.980 | 3370.1318 | 11565.5962 | 29144.3647 | 6.040 | 112 | 0.00000 | TRUE |
| Neuron-specific enolase (CSF) | MUO vs Controls | 16.685 | 9.1005 | -7.0463 | 40.4158 | 1.833 | 113 | 0.26320 | FALSE |
| Amyloid β1-42 (CSF) | CM/SM/M vs Controls | -12.855 | 106.9110 | -290.5753 | 264.8656 | -0.120 | 152 | 0.99938 | FALSE |
| Total tau (CSF) | CM/SM/M vs Controls | 17.197 | 9.0761 | -6.3760 | 40.7708 | 1.895 | 154 | 0.23454 | FALSE |
| Neurofilament-light chain (CSF) | CM/SM/M vs Controls | 10046.079 | 3853.8931 | -4.9666 | 20097.1242 | 2.607 | 112 | 0.05016 | FALSE |
| Neuron-specific enolase (CSF) | CM/SM/M vs Controls | 15.290 | 10.2304 | -11.3879 | 41.9671 | 1.495 | 113 | 0.44418 | FALSE |
| Amyloid β1-42 (CSF) | Tumors vs Controls | -577.492 | 121.7792 | -893.8353 | -261.1487 | -4.742 | 152 | 0.00003 | TRUE |
| Total tau (CSF) | Tumors vs Controls | 37.145 | 10.5998 | 9.6144 | 64.6761 | 3.504 | 154 | 0.00333 | TRUE |
| Neurofilament-light chain (CSF) | Tumors vs Controls | 4781.133 | 4002.1736 | -5656.6315 | 15218.8974 | 1.195 | 112 | 0.63151 | FALSE |
| Neuron-specific enolase (CSF) | Tumors vs Controls | 52.083 | 10.8072 | 23.9018 | 80.2650 | 4.819 | 113 | 0.00003 | TRUE |
| Amyloid β1-42 (CSF) | MUO vs CM/SM/M | -223.583 | 113.6698 | -518.8604 | 71.6949 | -1.967 | 152 | 0.20514 | FALSE |
| Total tau (CSF) | MUO vs CM/SM/M | 34.268 | 9.6783 | 9.1304 | 59.4056 | 3.541 | 154 | 0.00294 | TRUE |
| Neurofilament-light chain (CSF) | MUO vs CM/SM/M | 10308.902 | 4180.1340 | -592.9878 | 21210.7911 | 2.466 | 112 | 0.07092 | FALSE |
| Neuron-specific enolase (CSF) | MUO vs CM/SM/M | 1.395 | 11.1253 | -27.6161 | 30.4063 | 0.125 | 113 | 0.99929 | FALSE |
| Amyloid β1-42 (CSF) | MUO vs Tumors | 341.054 | 127.7538 | 9.1909 | 672.9178 | 2.670 | 152 | 0.04147 | TRUE |
| Total tau (CSF) | MUO vs Tumors | 14.320 | 11.1198 | -14.5613 | 43.2017 | 1.288 | 154 | 0.57208 | FALSE |
| Neurofilament-light chain (CSF) | MUO vs Tumors | 15573.848 | 4317.2238 | 4314.4245 | 26833.2705 | 3.607 | 112 | 0.00258 | TRUE |
| Neuron-specific enolase (CSF) | MUO vs Tumors | -35.399 | 11.6579 | -65.7988 | -4.9986 | -3.036 | 113 | 0.01552 | TRUE |
| Amyloid β1-42 (CSF) | CM/SM/M vs Tumors | 564.637 | 139.8258 | 201.4147 | 927.8596 | 4.038 | 152 | 0.00049 | TRUE |
| Total tau (CSF) | CM/SM/M vs Tumors | -19.948 | 11.9960 | -51.1050 | 11.2094 | -1.663 | 154 | 0.34692 | FALSE |
| Neurofilament-light chain (CSF) | CM/SM/M vs Tumors | 5264.946 | 4704.5856 | -7004.7259 | 17534.6176 | 1.119 | 112 | 0.67856 | FALSE |
| Neuron-specific enolase (CSF) | CM/SM/M vs Tumors | -36.794 | 12.5598 | -69.5458 | -4.0418 | -2.929 | 113 | 0.02110 | TRUE |

Supplementary Table 3: CSF biomarkers by Diagnostic Group comparison. **Age Covariate adjustment**

| **protein** | **contrast** | **mean diff** | **sd diff** | **lower** | **upper** | **t-statistic** | **df** | **p-value** | **significant** |
| --- | --- | --- | --- | --- | --- | --- | --- | --- | --- |
| Amyloid β1-42 (CSF) | MUO vs Controls | -213.345 | 89.6877 | -446.3419 | 19.6521 | -2.379 | 151 | 0.08558 | FALSE |
| Total tau (CSF) | MUO vs Controls | 51.725 | 7.9805 | 30.9958 | 72.4544 | 6.481 | 153 | 0.00000 | TRUE |
| Neurofilament-light chain (CSF) | MUO vs Controls | 19786.108 | 3412.6525 | 10884.6162 | 28687.5991 | 5.798 | 111 | 0.00000 | TRUE |
| Neuron-specific enolase (CSF) | MUO vs Controls | 14.807 | 9.3290 | -9.5234 | 39.1373 | 1.587 | 112 | 0.39006 | FALSE |
| Amyloid β1-42 (CSF) | CM/SM/M vs Controls | -48.392 | 106.3156 | -324.5857 | 227.8024 | -0.455 | 151 | 0.96853 | FALSE |
| Total tau (CSF) | CM/SM/M vs Controls | 16.860 | 9.2130 | -7.0705 | 40.7908 | 1.830 | 153 | 0.26328 | FALSE |
| Neurofilament-light chain (CSF) | CM/SM/M vs Controls | 10365.822 | 3864.5479 | 285.6157 | 20446.0278 | 2.682 | 111 | 0.04137 | TRUE |
| Neuron-specific enolase (CSF) | CM/SM/M vs Controls | 16.119 | 10.2759 | -10.6806 | 42.9189 | 1.569 | 112 | 0.40066 | FALSE |
| Amyloid β1-42 (CSF) | Tumors vs Controls | -487.312 | 125.6757 | -813.8017 | -160.8231 | -3.878 | 151 | 0.00090 | TRUE |
| Total tau (CSF) | Tumors vs Controls | 37.952 | 11.1571 | 8.9718 | 66.9326 | 3.402 | 153 | 0.00470 | TRUE |
| Neurofilament-light chain (CSF) | Tumors vs Controls | 3158.616 | 4292.2863 | -8037.2941 | 14354.5256 | 0.736 | 111 | 0.88243 | FALSE |
| Neuron-specific enolase (CSF) | Tumors vs Controls | 48.049 | 11.6581 | 17.6440 | 78.4532 | 4.121 | 112 | 0.00042 | TRUE |
| Amyloid β1-42 (CSF) | MUO vs CM/SM/M | -164.953 | 114.5719 | -462.5961 | 132.6896 | -1.440 | 151 | 0.47669 | FALSE |
| Total tau (CSF) | MUO vs CM/SM/M | 34.865 | 10.0250 | 8.8249 | 60.9048 | 3.478 | 153 | 0.00365 | TRUE |
| Neurofilament-light chain (CSF) | MUO vs CM/SM/M | 9420.286 | 4264.4247 | -1702.9504 | 20543.5222 | 2.209 | 111 | 0.12712 | FALSE |
| Neuron-specific enolase (CSF) | MUO vs CM/SM/M | -1.312 | 11.5095 | -31.3292 | 28.7049 | -0.114 | 112 | 0.99947 | FALSE |
| Amyloid β1-42 (CSF) | MUO vs Tumors | 273.968 | 128.8747 | -60.8323 | 608.7673 | 2.126 | 151 | 0.14961 | FALSE |
| Total tau (CSF) | MUO vs Tumors | 13.773 | 11.3873 | -15.8057 | 43.3514 | 1.209 | 153 | 0.62179 | FALSE |
| Neurofilament-light chain (CSF) | MUO vs Tumors | 16627.492 | 4432.1351 | 5066.8034 | 28188.1805 | 3.752 | 111 | 0.00158 | TRUE |
| Neuron-specific enolase (CSF) | MUO vs Tumors | -33.242 | 11.8954 | -64.2650 | -2.2183 | -2.795 | 112 | 0.03069 | TRUE |
| Amyloid β1-42 (CSF) | CM/SM/M vs Tumors | 438.921 | 147.3301 | 56.1762 | 821.6653 | 2.979 | 151 | 0.01755 | TRUE |
| Total tau (CSF) | CM/SM/M vs Tumors | -21.092 | 12.9527 | -54.7367 | 12.5526 | -1.628 | 153 | 0.36580 | FALSE |
| Neurofilament-light chain (CSF) | CM/SM/M vs Tumors | 7207.206 | 5057.8612 | -5985.6134 | 20400.0255 | 1.425 | 111 | 0.48658 | FALSE |
| Neuron-specific enolase (CSF) | CM/SM/M vs Tumors | -31.929 | 13.6205 | -67.4521 | 3.5932 | -2.344 | 112 | 0.09429 | FALSE |

Supplementary Table 4: CSF biomarkers by Diagnostic Group comparison. **Sex Covariate adjustment**

| **protein** | **contrast** | **mean diff** | **sd diff** | **lower** | **upper** | **t-statistic** | **df** | **p-value** | **significant** |
| --- | --- | --- | --- | --- | --- | --- | --- | --- | --- |
| Amyloid β1-42 (CSF) | MUO vs Controls | -233.272 | 91.9810 | -472.2625 | 5.7189 | -2.536 | 149 | 0.05852 | FALSE |
| Total tau (CSF) | MUO vs Controls | 50.777 | 7.9598 | 30.0981 | 71.4551 | 6.379 | 151 | 0.00000 | TRUE |
| Neurofilament-light chain (CSF) | MUO vs Controls | 21113.329 | 3485.3046 | 12019.7888 | 30206.8701 | 6.058 | 109 | 0.00000 | TRUE |
| Neuron-specific enolase (CSF) | MUO vs Controls | 17.412 | 9.3281 | -6.9228 | 41.7462 | 1.867 | 110 | 0.24847 | FALSE |
| Amyloid β1-42 (CSF) | CM/SM/M vs Controls | -14.047 | 107.7468 | -294.0012 | 265.9072 | -0.130 | 149 | 0.99921 | FALSE |
| Total tau (CSF) | CM/SM/M vs Controls | 17.443 | 9.1191 | -6.2475 | 41.1330 | 1.913 | 151 | 0.22704 | FALSE |
| Neurofilament-light chain (CSF) | CM/SM/M vs Controls | 10056.191 | 3889.7510 | -92.5949 | 20204.9762 | 2.585 | 109 | 0.05309 | FALSE |
| Neuron-specific enolase (CSF) | CM/SM/M vs Controls | 14.813 | 10.2979 | -12.0514 | 41.6777 | 1.438 | 110 | 0.47829 | FALSE |
| Amyloid β1-42 (CSF) | Tumors vs Controls | -592.435 | 124.0120 | -914.6505 | -270.2193 | -4.777 | 149 | 0.00002 | TRUE |
| Total tau (CSF) | Tumors vs Controls | 38.872 | 10.7321 | 10.9919 | 66.7530 | 3.622 | 151 | 0.00223 | TRUE |
| Neurofilament-light chain (CSF) | Tumors vs Controls | 4843.779 | 4107.8244 | -5873.9838 | 15561.5420 | 1.179 | 109 | 0.64126 | FALSE |
| Neuron-specific enolase (CSF) | Tumors vs Controls | 49.994 | 11.0644 | 21.1298 | 78.8580 | 4.518 | 110 | 0.00009 | TRUE |
| Amyloid β1-42 (CSF) | MUO vs CM/SM/M | -219.225 | 115.0147 | -518.0630 | 79.6133 | -1.906 | 149 | 0.22991 | FALSE |
| Total tau (CSF) | MUO vs CM/SM/M | 33.334 | 9.7404 | 8.0297 | 58.6380 | 3.422 | 151 | 0.00440 | TRUE |
| Neurofilament-light chain (CSF) | MUO vs CM/SM/M | 11057.139 | 4285.8825 | -125.1971 | 22239.4747 | 2.580 | 109 | 0.05381 | FALSE |
| Neuron-specific enolase (CSF) | MUO vs CM/SM/M | 2.599 | 11.3549 | -27.0235 | 32.2206 | 0.229 | 110 | 0.99575 | FALSE |
| Amyloid β1-42 (CSF) | MUO vs Tumors | 359.163 | 130.6113 | 19.8009 | 698.5253 | 2.750 | 149 | 0.03353 | TRUE |
| Total tau (CSF) | MUO vs Tumors | 11.904 | 11.2958 | -17.4408 | 41.2491 | 1.054 | 151 | 0.71799 | FALSE |
| Neurofilament-light chain (CSF) | MUO vs Tumors | 16269.550 | 4484.5319 | 4568.9165 | 27970.1842 | 3.628 | 109 | 0.00243 | TRUE |
| Neuron-specific enolase (CSF) | MUO vs Tumors | -32.582 | 12.0154 | -63.9273 | -1.2371 | -2.712 | 110 | 0.03835 | TRUE |
| Amyloid β1-42 (CSF) | CM/SM/M vs Tumors | 578.388 | 141.8303 | 209.8759 | 946.8999 | 4.078 | 149 | 0.00042 | TRUE |
| Total tau (CSF) | CM/SM/M vs Tumors | -21.430 | 12.0891 | -52.8355 | 9.9761 | -1.773 | 151 | 0.29055 | FALSE |
| Neurofilament-light chain (CSF) | CM/SM/M vs Tumors | 5212.412 | 4788.1006 | -7280.2663 | 17705.0893 | 1.089 | 109 | 0.69722 | FALSE |
| Neuron-specific enolase (CSF) | CM/SM/M vs Tumors | -35.181 | 12.7602 | -68.4688 | -1.8928 | -2.757 | 110 | 0.03401 | TRUE |

Supplementary Table 5: CSF biomarkers by Diagnostic Group comparison. **Castration Covariate adjustment**

| **protein** | **contrast** | **mean diff** | **sd diff** | **lower** | **upper** | **t-statistic** | **df** | **p-value** | **significant** |
| --- | --- | --- | --- | --- | --- | --- | --- | --- | --- |
| Amyloid β1-42 (CSF) | MUO vs Controls | -235.537 | 91.5552 | -473.3853 | 2.3116 | -2.573 | 151 | 0.05333 | FALSE |
| Total tau (CSF) | MUO vs Controls | 50.976 | 7.9476 | 30.3318 | 71.6194 | 6.414 | 153 | 0.00000 | TRUE |
| Neurofilament-light chain (CSF) | MUO vs Controls | 21057.680 | 3454.8927 | 12046.0102 | 30069.3502 | 6.095 | 111 | 0.00000 | TRUE |
| Neuron-specific enolase (CSF) | MUO vs Controls | 17.517 | 9.3022 | -6.7433 | 41.7775 | 1.883 | 112 | 0.24116 | FALSE |
| Amyloid β1-42 (CSF) | CM/SM/M vs Controls | -12.717 | 107.2763 | -291.4066 | 265.9730 | -0.119 | 151 | 0.99940 | FALSE |
| Total tau (CSF) | CM/SM/M vs Controls | 17.020 | 9.1019 | -6.6220 | 40.6621 | 1.870 | 153 | 0.24534 | FALSE |
| Neurofilament-light chain (CSF) | CM/SM/M vs Controls | 10056.890 | 3856.0969 | -1.2732 | 20115.0523 | 2.608 | 111 | 0.05004 | FALSE |
| Neuron-specific enolase (CSF) | CM/SM/M vs Controls | 15.237 | 10.2664 | -11.5376 | 42.0126 | 1.484 | 112 | 0.45042 | FALSE |
| Amyloid β1-42 (CSF) | Tumors vs Controls | -577.271 | 122.2108 | -894.7592 | -259.7834 | -4.724 | 151 | 0.00003 | TRUE |
| Total tau (CSF) | Tumors vs Controls | 37.068 | 10.6244 | 9.4712 | 64.6646 | 3.489 | 153 | 0.00351 | TRUE |
| Neurofilament-light chain (CSF) | Tumors vs Controls | 4763.115 | 4004.4906 | -5682.1147 | 15208.3447 | 1.189 | 111 | 0.63478 | FALSE |
| Neuron-specific enolase (CSF) | Tumors vs Controls | 52.059 | 10.8448 | 23.7757 | 80.3425 | 4.800 | 112 | 0.00003 | TRUE |
| Amyloid β1-42 (CSF) | MUO vs CM/SM/M | -222.820 | 114.4468 | -520.1381 | 74.4980 | -1.947 | 151 | 0.21307 | FALSE |
| Total tau (CSF) | MUO vs CM/SM/M | 33.956 | 9.7160 | 8.7184 | 59.1927 | 3.495 | 153 | 0.00344 | TRUE |
| Neurofilament-light chain (CSF) | MUO vs CM/SM/M | 11000.791 | 4247.5508 | -78.4321 | 22080.0134 | 2.590 | 111 | 0.05238 | FALSE |
| Neuron-specific enolase (CSF) | MUO vs CM/SM/M | 2.280 | 11.3215 | -27.2470 | 31.8062 | 0.201 | 112 | 0.99709 | FALSE |
| Amyloid β1-42 (CSF) | MUO vs Tumors | 341.734 | 128.4592 | 8.0141 | 675.4548 | 2.660 | 151 | 0.04253 | TRUE |
| Total tau (CSF) | MUO vs Tumors | 13.908 | 11.1689 | -15.1035 | 42.9190 | 1.245 | 153 | 0.59915 | FALSE |
| Neurofilament-light chain (CSF) | MUO vs Tumors | 16294.565 | 4388.0016 | 4848.9936 | 27740.1369 | 3.713 | 111 | 0.00180 | TRUE |
| Neuron-specific enolase (CSF) | MUO vs Tumors | -34.542 | 11.8395 | -65.4198 | -3.6642 | -2.918 | 112 | 0.02186 | TRUE |
| Amyloid β1-42 (CSF) | CM/SM/M vs Tumors | 564.554 | 140.2889 | 200.1020 | 929.0070 | 4.024 | 151 | 0.00052 | TRUE |
| Total tau (CSF) | CM/SM/M vs Tumors | -20.048 | 12.0241 | -51.2803 | 11.1847 | -1.667 | 153 | 0.34456 | FALSE |
| Neurofilament-light chain (CSF) | CM/SM/M vs Tumors | 5293.775 | 4707.3558 | -6984.7940 | 17572.3431 | 1.125 | 111 | 0.67520 | FALSE |
| Neuron-specific enolase (CSF) | CM/SM/M vs Tumors | -36.822 | 12.6035 | -69.6918 | -3.9514 | -2.922 | 112 | 0.02161 | TRUE |

Supplementary Table 6: Intercorrelation of biomarkers by diagnosis (details)

| **diagnosis** | **variable** | **n** | **r** | **lower** | **upper** | **test-statistic** | **p-value** | **significant** | **direction** |
| --- | --- | --- | --- | --- | --- | --- | --- | --- | --- |
| MUO | Amyloid β1-42 (CSF) and Total tau (CSF) | 42 | 0.025 | -0.2806 | 0.3269 | 0.159 | 0.87359 | FALSE |  |
| MUO | Amyloid β1-42 (CSF) and Neurofilament-light chain (CSF) | 29 | 0.072 | -0.3023 | 0.4274 | 0.369 | 0.71208 | FALSE |  |
| MUO | Amyloid β1-42 (CSF) and Neuron-specific enolase (CSF) | 29 | -0.019 | -0.3827 | 0.3501 | -0.096 | 0.92362 | FALSE |  |
| MUO | Total tau (CSF) and Neurofilament-light chain (CSF) | 29 | 0.606 | 0.3086 | 0.7960 | 3.586 | 0.00034 | TRUE | plus |
| MUO | Total tau (CSF) and Neuron-specific enolase (CSF) | 30 | 0.742 | 0.5217 | 0.8700 | 4.967 | 0.00000 | TRUE | plus |
| MUO | Neurofilament-light chain (CSF) and Neuron-specific enolase (CSF) | 29 | 0.519 | 0.1887 | 0.7441 | 2.934 | 0.00335 | TRUE | plus |
| CM/SM/M | Amyloid β1-42 (CSF) and Total tau (CSF) | 27 | -0.133 | -0.4887 | 0.2597 | -0.658 | 0.51074 | FALSE |  |
| CM/SM/M | Amyloid β1-42 (CSF) and Neurofilament-light chain (CSF) | 19 | -0.183 | -0.5886 | 0.2954 | -0.742 | 0.45800 | FALSE |  |
| CM/SM/M | Amyloid β1-42 (CSF) and Neuron-specific enolase (CSF) | 20 | 0.194 | -0.2716 | 0.5864 | 0.811 | 0.41725 | FALSE |  |
| CM/SM/M | Total tau (CSF) and Neurofilament-light chain (CSF) | 20 | 0.855 | 0.6633 | 0.9413 | 5.253 | 0.00000 | TRUE | plus |
| CM/SM/M | Total tau (CSF) and Neuron-specific enolase (CSF) | 21 | 0.088 | -0.3571 | 0.5008 | 0.375 | 0.70760 | FALSE |  |
| CM/SM/M | Neurofilament-light chain (CSF) and Neuron-specific enolase (CSF) | 20 | 0.055 | -0.3975 | 0.4854 | 0.225 | 0.82179 | FALSE |  |
| Tumors | Amyloid β1-42 (CSF) and Total tau (CSF) | 19 | -0.324 | -0.6783 | 0.1529 | -1.344 | 0.17910 | FALSE |  |
| Tumors | Amyloid β1-42 (CSF) and Neurofilament-light chain (CSF) | 18 | -0.158 | -0.5819 | 0.3336 | -0.617 | 0.53749 | FALSE |  |
| Tumors | Amyloid β1-42 (CSF) and Neuron-specific enolase (CSF) | 18 | -0.287 | -0.6651 | 0.2073 | -1.145 | 0.25202 | FALSE |  |
| Tumors | Total tau (CSF) and Neurofilament-light chain (CSF) | 18 | 0.660 | 0.2797 | 0.8616 | 3.073 | 0.00212 | TRUE | plus |
| Tumors | Total tau (CSF) and Neuron-specific enolase (CSF) | 18 | 0.488 | 0.0273 | 0.7777 | 2.066 | 0.03886 | TRUE | plus |
| Tumors | Neurofilament-light chain (CSF) and Neuron-specific enolase (CSF) | 18 | 0.583 | 0.1593 | 0.8252 | 2.582 | 0.00982 | TRUE | plus |
| Controls | Amyloid β1-42 (CSF) and Total tau (CSF) | 64 | 0.070 | -0.1791 | 0.3103 | 0.546 | 0.58495 | FALSE |  |
| Controls | Amyloid β1-42 (CSF) and Neurofilament-light chain (CSF) | 48 | -0.324 | -0.5567 | -0.0436 | -2.253 | 0.02427 | TRUE | minus |
| Controls | Amyloid β1-42 (CSF) and Neuron-specific enolase (CSF) | 48 | 0.262 | -0.0242 | 0.5081 | 1.798 | 0.07225 | FALSE |  |
| Controls | Total tau (CSF) and Neurofilament-light chain (CSF) | 48 | 0.209 | -0.0799 | 0.4655 | 1.423 | 0.15473 | FALSE |  |
| Controls | Total tau (CSF) and Neuron-specific enolase (CSF) | 48 | 0.063 | -0.2254 | 0.3408 | 0.422 | 0.67329 | FALSE |  |
| Controls | Neurofilament-light chain (CSF) and Neuron-specific enolase (CSF) | 47 | 0.371 | 0.0937 | 0.5947 | 2.583 | 0.00979 | TRUE | plus |

# MRI examples

## MRI scan of MUO


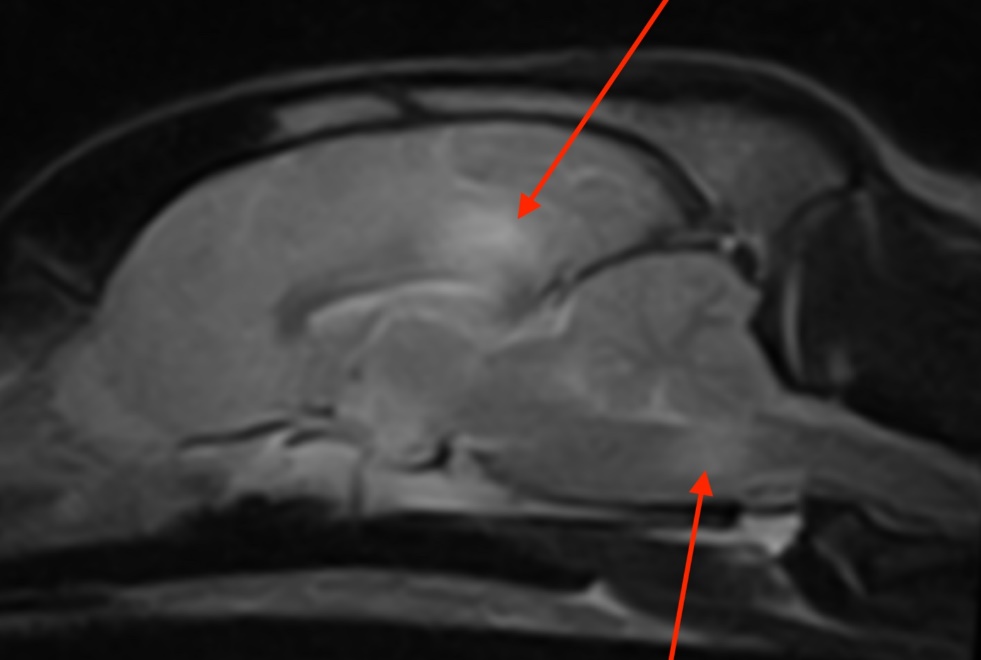


**Supplementary Figure 1: Multifocal MUO with hyperintense lesions in brain stem and neocortex.** Multiple diffuse T2 hyperintense, T1 isointense lesions with mild saturation postcontrast are present predominantly in the left parietal and occipital lobe and brainstem (red arrows).

## MRI scan of a Tumor

*
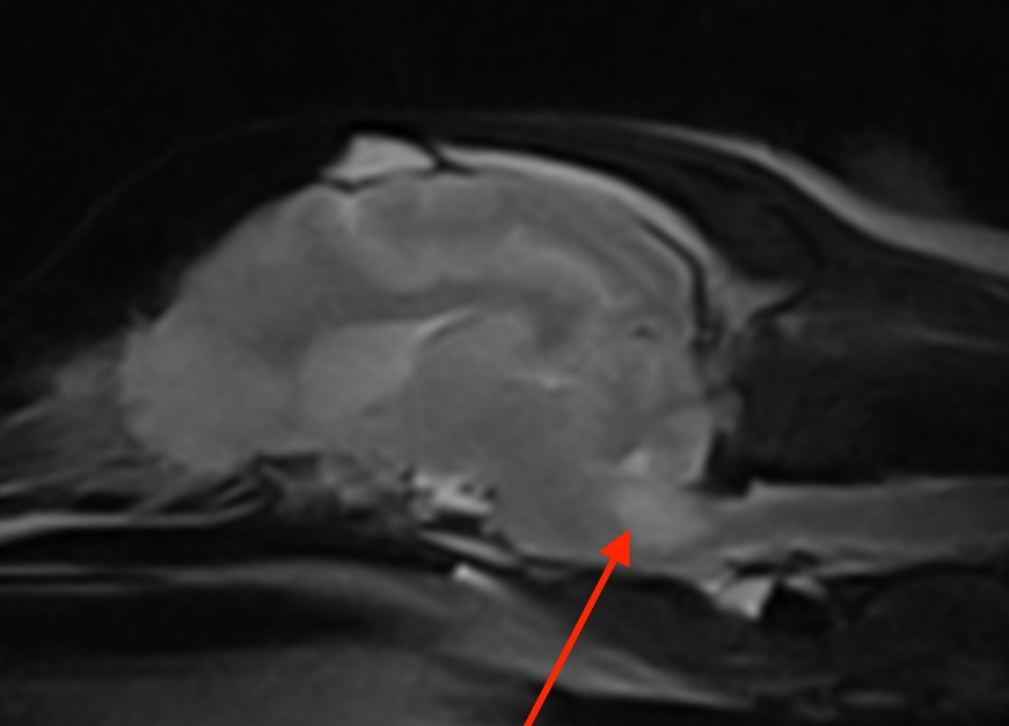
*

**Supplementary Figure 2: Suspected glioma in the brain stem.** A T2 hyperintense, T1 isointense lesion is observed on the right side of the brainstem. The lesion displays a mass effect and saturation postcontrast.

## Equivocal MRI (differential diagnosis – tumor vs. MUO)


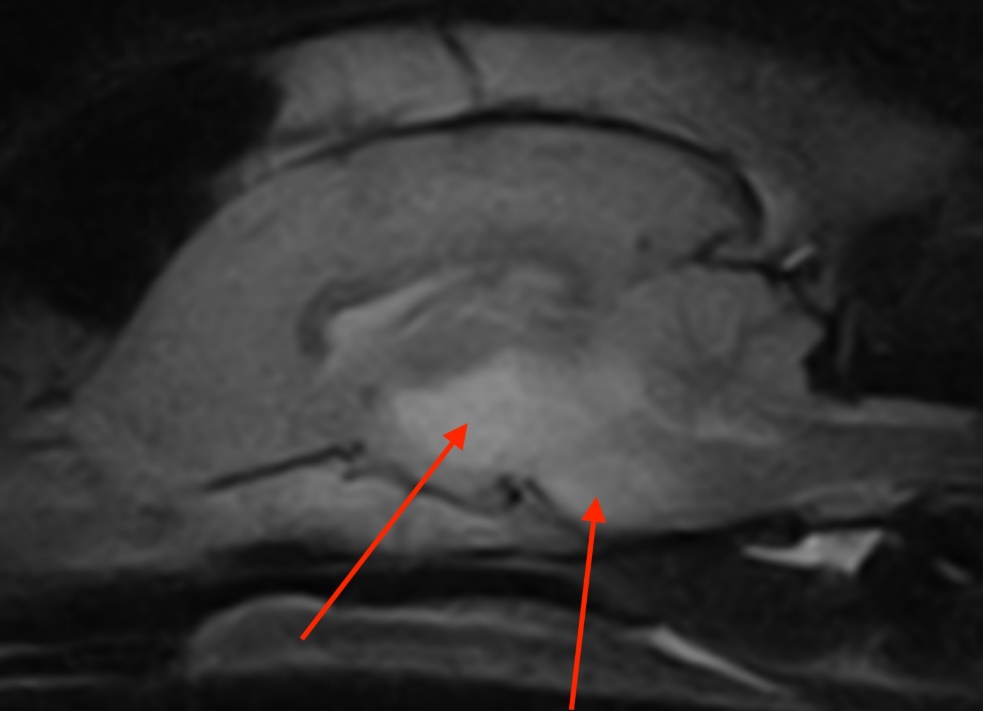


**Supplementary Figure 3:** Differential diagnosis: glioma vs. MUO. A right-sided diffuse intra-axial, expansive, T2 hyperintense, T1 isointense lesion in the area of the thalamus is trespassing on the brainstem. The lesion does not display saturation postcontrast in the T1 sequence.

## MRI scan of Syringomyelia and Chiari malformation

*
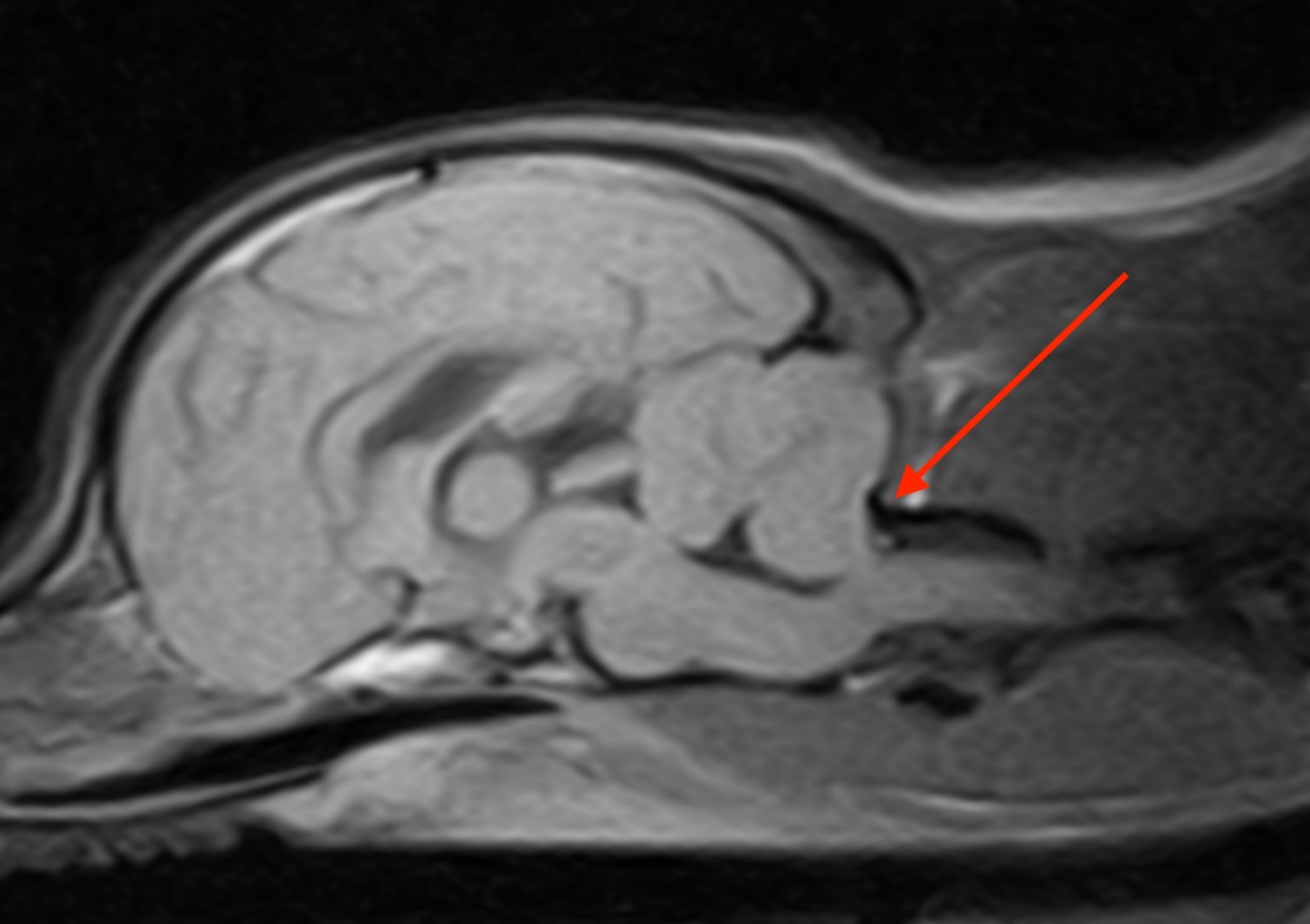
*

**Supplementary Figure 4: Syringomyelia with Chiari malformation.** The MRI of the brain and cervical spinal cord shows a caudal malformation of the occipital bone (Chiari-like malformation), herniation and compression of the cerebellum against the brainstem. MRI cervical segments and spinal cord showed also syringomyelia.

## MRI scan of Myelitis

*
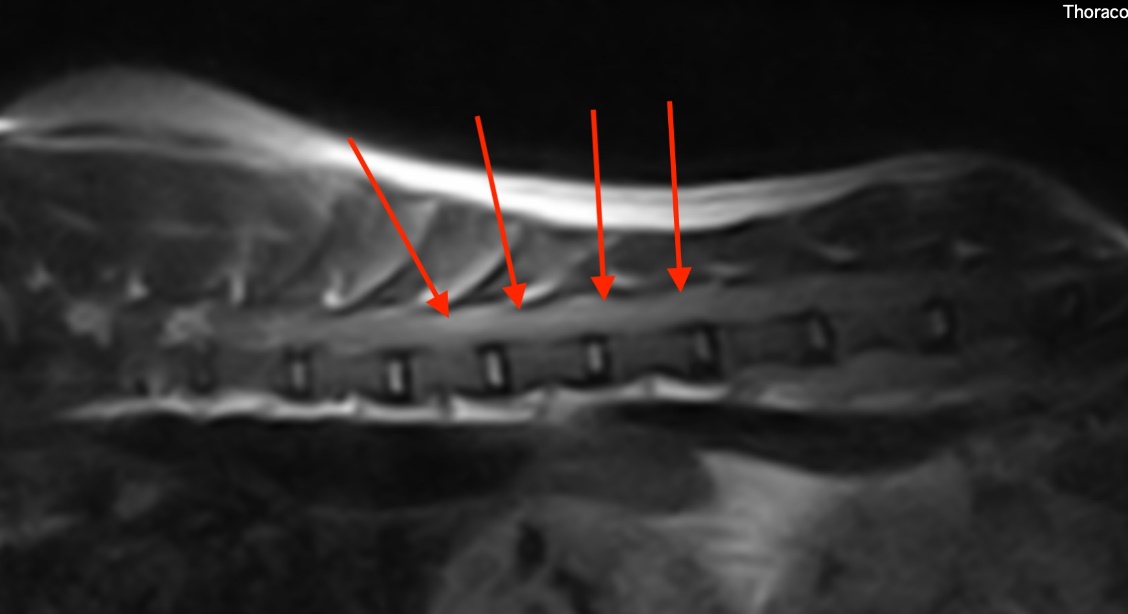
*

**Supplementary Figure 5: Sagittal T2-weighted MRI scan of the thoraco-lumbar spine and spinal cord.** Arrows indicate a diffuse, T2 and STIR hyperintense, intra-medullar lesion at the T12-T13-L1 level. In T1-weighted images, a massive saturation postcontrast is observed in the meninges.

# Dogs breeds by diagnosis

Supplementary Table 7: Dog breeds by diagnosis

| **diagnosis** | **breed** | **n** |
| --- | --- | --- |
| MUO | Yorkshire Terrier | 13 |
| MUO | Mix | 5 |
| MUO | Pug | 4 |
| MUO | French Bulldog | 3 |
| MUO | Jack Russell Terrier | 3 |
| MUO | Chihuahua | 2 |
| MUO | Maltese | 2 |
| MUO | Spitz | 2 |
| MUO | American Staffordshire Terrier | 1 |
| MUO | Boston Terrier | 1 |
| MUO | Dachshund | 1 |
| MUO | English Springer Spaniel | 1 |
| MUO | Griffon | 1 |
| MUO | Hungarian Vizsla | 1 |
| MUO | Lhasa Apso | 1 |
| MUO | Papillon | 1 |
| MUO | Pomeranian | 1 |
| MUO | Prague Ratter | 1 |
| MUO | Siberian Husky | 1 |
| MUO | Weimaraner | 1 |
| MUO | Whippet | 1 |
| CM/SM/M | French Bulldog | 8 |
| CM/SM/M | Cavalier King Charles Spaniel | 5 |
| CM/SM/M | Maltese | 2 |
| CM/SM/M | Mix | 2 |
| CM/SM/M | Pug | 2 |
| CM/SM/M | Yorkshire Terrier | 2 |
| CM/SM/M | Biewer Terrier | 1 |
| CM/SM/M | Bolognese | 1 |
| CM/SM/M | Border Terrier | 1 |
| CM/SM/M | Chihuahua | 1 |
| CM/SM/M | English Bulldog | 1 |
| CM/SM/M | Miniature Pinscher | 1 |
| CM/SM/M | Shi-Tzu | 1 |
| CM/SM/M | West Highland White Terrier | 1 |
| Tumors | Mix | 6 |
| Tumors | Yorkshire Terrier | 4 |
| Tumors | American Staffordshire Terrier | 2 |
| Tumors | Jack Russell Terrier | 2 |
| Tumors | Boston Terrier | 1 |
| Tumors | Chihuahua | 1 |
| Tumors | English Cocker Spaniel | 1 |
| Tumors | Maltese | 1 |
| Tumors | Rhodesian Ridgeback | 1 |
| Controls | Mix | 9 |
| Controls | Labrador Retriever | 7 |
| Controls | Yorkshire Terrier | 5 |
| Controls | Border Collie | 3 |
| Controls | Cane Corso | 3 |
| Controls | Chihuahua | 3 |
| Controls | German Pointer | 3 |
| Controls | Hungarian Vizsla | 3 |
| Controls | Maltese | 3 |
| Controls | Beagle | 2 |
| Controls | Akita Inu | 1 |
| Controls | Alaskan Malamut | 1 |
| Controls | American Staffordshire Terrier | 1 |
| Controls | Australian Sheperd Dog | 1 |
| Controls | Belgian Shepard | 1 |
| Controls | Bichon Frise | 1 |
| Controls | Bobtail | 1 |
| Controls | Bolognese | 1 |
| Controls | Boston Terrier | 1 |
| Controls | Boxer | 1 |
| Controls | Chinese Crested Dog | 1 |
| Controls | English Bulldog | 1 |
| Controls | English Cocker Spaniel | 1 |
| Controls | French Bulldog | 1 |
| Controls | Italian Greyhound | 1 |
| Controls | Jack Russell Terrier | 1 |
| Controls | Lagotto Romagnolo | 1 |
| Controls | Lhasa Apso | 1 |
| Controls | Moscow Watchdog | 1 |
| Controls | Pekingese | 1 |
| Controls | Rhodesian Ridgeback | 1 |
| Controls | Siberian Husky | 1 |
| Controls | Staffordshire Bull Terrier | 1 |
| Controls | Toy Poodle | 1 |
| Controls | Whippet | 1 |

# Clinical, MRI, and CSF analysis details for individual animals

Supplementary Table 8: Clinical, MRI, and CSF analysis details for individual animals

| Animal ID | Clinical presentation | Imaging findings | CSF | Seizures | Diagnosis |
| --- | --- | --- | --- | --- | --- |
| 81D | Drooping jaw, inability to feed, weak palpebral reflex | MRI: Normal | Pure, Pandy's test NA, mononuclear pleocytosis 14 cells/µL, presence of activated macrophages | no | MUO |
| 58D | Head trauma, painful right eye, motor deficits | MRI: Multiple T2 hyperintense, T1 isointense lesions with mild saturation postcontrast. Lesions present predominantly in the left parietal and occipital lobe and brainstem | Pure, Pandy's test NA, severe mononuclear pleocytosis 152 cells/µL with presence of activated macrophages | no | MUO |
| 50D | Apathy, somnolence, motor deficits, circling to the left | MRI: Diffuse intra-axial , expansive, T2 hyperintense, T1 isointense lesion in Thalamus area on the right side trespassing to the brainstem, lesion without saturation postcontrast on T1 sequence | Pure, Pandy's test NA, mononuclear pleocytosis 34 cells/µL, presence of activated macrophages, mild blood contamination | no | MUO |
| 31D | Motor deficits, cervical hyperaesthesia | MRI: Diffuse, T2 hyperintense, intramedullar lesion in spinal cord. Multiple, diffuse , T2 hyperintense,T1 isointense lesions predominantly present in Thalamus, brainstem and left parietal lobe. Lesions were saturated postcontrast | Pure, Pandy's test NA, mononuclear pleocytosis 8 cells/µL with presence of activated macrophages | no | MUO |
| 22D | Seizures | MRI: Multiple, diffuse, intra-axial, T2 hyperintense and T1 isointense lesions without saturation postcontrast, no mass effect and predominatly present in white matter of prosencephalon | Pure, Pandy's test NA, mononuclear pleocytosis 9 cells/µL | yes | MUO |
| 20D | Sudden blindness for 3 hours, no symptoms thereafter | MRI: Normal | Pure, Pandy's test NA, mononuclear pleocytosis 15 cells/µL | no | MUO |
| 10D | Apathy, circling to the left, weak pupilar reflex, hyperreflexia and hypermetry on all limbs. | MRI: Multiple diffuse intra-axial, T2 hyperintense and T1 hypointense mass lesions with saturation postcontrast. Lesions were visible everywhere, but predominantly in brainstem and diencephalon | Pure, Pandy's test NA, mononuclear pleocytosis 30 cells/µL | no | MUO |
| 2D | Seizures, circling to the left, ataxia and hypereflexia on all 4 limbs, spinal hyperaesthesia | MRI: Multiple diffuse T2 hyperintense and hypointense lesions present throughout prosencephalon with saturation postcontrast | Pure, Pandy's test NA, mononuclear pleocytosis 25 cells/µL with activated macrophages | yes | MUO |
| 3D | Blindness, motor deficits, quadriplegia, cervical hyperaesthesia | MRI: Ventriculomegalia, othervise normal. Chronic protrusions of intervertebral disc C6-C7 with compresion of spinal cord. Over C6-C7 protrusion T2 hyperintense intramedullar lesion was detected | Pure, Pandy's test NA, mononuclear pleocytosis 25 cells/µL with activated macrophages | no | M |
| 6D | Motor deficits, quadriplegia, hyperreflexia on hind limbs, hyporeflexia on forelimbs, mild pain in cervical area. | MRI: Multiple degeneration on intervertebral discs in cervical area without compression of spinal cord. Protrusion C4-C7 with mild compression of spinal cord. Intramedullar lesion in CC5-C6 with saturation postcontrast. Saturation also seen in radices of nerves in C4-C6 segments | Pure, Pandy's test NA, mononuclear pleocytosis 20 cells/µL | no | M |
| 60D | Bilateral lateral strabism, horizontal nystagmus, partial blindness, disorientation | MRI: Diffuse T2 hyperintense and T1 hypointense lesions with mild saturation postcontrast. Lesions predominantly present in white matter of prosencephalon | Pure, Pandy's test NA, severe mononuclear pleocytosis 82 cells/µL with activated macrophages | no | MUO |
| 36D | Disorientation, motor deficits, circling to the left | MRI: Brain multiple diffuse T2 hyperintense, T1 isointense to hypointense lesions without saturation postcontrast. Lesions present throughout the brain, with the most prominent lesion in brainstem | Pure, Pandy's test NA, mononuclear pleocytosis 54 cells/µL | no | MUO |
| 61D | Vestibular ataxia, no papebral reflex, motor deficits, ventral strabism on right side, head tremor | MRI: T2 hyperintense, T1 isointense to hypointense lesion with saturation postcontrast. Lesions present mainly in rostral cerebellum | Pure, Pandy's test NA, mononuclear pleocytosis 109 cells/µL | no | MUO |
| 71D | Ataxia, myoclonus, motor deficits, cystitis | MRI: Brain and spinal cord (cervical -lumbal segments) showed quadrigeminal cyst, occipital dysplasia, atlanto-occipital overlap (AO overlap), herniation of cerebellum and compressed brainstem, multiple degeneration of intervertebral discs without compression of spinal cord and massive syringomyelia | Pure, Pandy's test NA, mononuclear pleocytosis 20 cells/µL with activated macrophages | no | M |
| 83D | Paraplegia, kyphosis, cervical and lumbal hyperaesthesia | MRI: Normal. | Pure, Pandy's test NA, mononuclear pleocytosis 75 cells/µL with activated macrophages | no | MUO |
| 89D | Paraplegia, motor deficits, hyperreflexia on hind limbs and thoraco-lumbar hyperaesthesia | MRI: Thoraco-lumbar, lumbar and lumbo-sacral spinal cord showed diffuse , T2 hyperintense, intamedullar lesion in spinal cord. In segments T12-T13-L1 diffuse ,T2 and STIR hyperintense lesion and paraspinal muscles and peripheral nerves. On this level T1 masive saturation postcontrast on meninges | Pure, Pandy's test NA, mixed pleocytosis 215 cells/µL | no | M |
| 119D | Ataxia, motor deficits, tremor | MRI: Brain, cervical segments and spinal cord normal | Pure, Pandy's test NA, mononuclear pleocytosis 17 cells/µL | no | MUO |
| 121D | Ataxia, seizures, motor deficits | MRI: Bilateral symetric hyperintense lesions on T2 sequence in Thalamus, and diffuse saturation of leptomeninx on T1 sequence postcontrast | Pure, Pandy's test NA, mononuclear pleocytosis 30 cells/µL with activated macrophages | yes | MUO |
| 126D | Right hemiplegia, cervical hyperaesthesia, weak flexor reflex on righr forelimb | MRI: Brain normal, MRI cervical segments and spinal cord normal | Pure, Pandy's test NA, massive elevation of nuclear elements 21 cells/µL, mixed pleocytosis with activated macrophages | no | MUO |
| 143D | Motor deficits, paraplegia, hyperreflexia on hind limbs | MRI: Liquid content in the left tympanic bulb and epithelium of middle ear with saturation postcontrast. Thoraco-lumbar and lumbo-sacral segments of spinal cord showed multiple degeneration on intervertebral discs without compression of nerves. Analysis of T12-T13-L1-L2 segments showed presence of diffuse , intramedullar, T2 hyperintense lesion | Pure, Pandy's test NA, mononuclear pleocytosis 7 cells/µL with activated macrophages | no | M |
| 147D | Quadriplegia, motor deficits, impaired cervical flexibility | MRI: Normal. MRI of cervical segments and spinal cord showed diffuse , intramedullar , T2 hyperintense lesion at C2-C3-C4-C5-C6 level, most prominent in C5 segment | Pure, Pandy's test NA, massive mononuclear pleocytosis 76 cells/µL with activated macrophages | no | M |
| 149D | Ataxia, disorientation, circling to the right, ventromedial right strabism | MRI: Multiple diffuse T2 hyperintense lesions in caudate nucleus area without saturation postcontrast | Pure, Pandy's test NA, massive mixed pleocytosis 534 cells/µL with activated macrophages | no | MUO |
| 165D | Paraplegia, kyphosis, thoraco-lumbar hyperaesthesia | MRI: Thoraco -lumbar, lumbar, lumbo-sacral segments and spinal cord showed diffuse intraparenchymal T2 hyperintense lesion predominantly presented in thoraco-lumbar area | Pure, Pandy's test NA, mononuclear pleocytosis 70 cells/µL, presence of activated macrophages | no | M |
| 168D | Seizures, apathy | MRI: Diffuse T2 hyperintense , T1 hypointense lesions in prosencephalon without saturation postcontrast | Pure, Pandy's test NA, mononuclear pleocytosis 7 cells/µL, presence of activated macrophages | yes | MUO |
| 169D | Disorientation, tremor, agression, cervical hyperaesthesia | MRI: Brain normal, MRI cervical segments and spinal cord normal | Pure, Pandy's test NA, mononuclear pleocytosis 6 cells/µL, presence of activated macrophages | no | MUO |
| 170D | Seizures | MRI: Normal, cervical segments with multiple degeneration on intervertebral discs without compression of spinal cord | Pure, Pandy's test NA, mononuclear pleocytosis 15 cells/µL, presence of activated macrophages | yes | MUO |
| 178D | Periodontitis grade 3, cervical hyperaesthesia | MRI: Cervical segments and spinal cord showed degeneration on C6-C7 intervertebral disc without compression of the spinal cord | Pure, Pandy's test NA, mononuclear pleocytosis 8 cells/µL, presence of activated macrophages | no | M |
| 184D | Paraplegia, blindness, cataracts, mydriasis, motor deficits, thoraco-lumbar hyperaesthesia | MRI: Brain and spinal cord normal | Pure, Pandy's test NA, massive mononuclear pleocytosis 390 cells/µL, presence of activated macrophages | no | MUO |
| 116D | Bilateral cataracts, compulsive walking, falling to the right side, cervical hyperaesthesia, multiple lumps on mamillary glands | MRI: Diffuse T2 hyperintense lesions predominantly in prosencephalon and Thalamus | Pure, Pandy's test NA, mononuclear pleocytosis 19 cells/µL, presence of activated macrophages | no | MUO |
| 18 D | Weak muscle tone, heart murmur, ataxia | MRI: T2 hyperintense , T1 isointense lesion on the right side of brainstem. Lesion presented with mass effect and saturation postcontrast | Pure, Pandy's test NA, mononuclear pleocytosis 10 cells/µL, presence of activated macrophages | no | Tumor |
| 17D | Ataxia, quadriplegia, cervical hyperaesthesia, lateral flexion to the left | MRI: Brain and cervical segments of the spinal cord showed multiple degeneration on intervertebral discs without spinal cord compression. Cervical segments and spinal cord showed caudal malformation of occipital bone (Chiari-like malformation) and extensive syringomyelia | Pure, Pandy's test NA, no pleocytosis | no | CM/SM |
| 69D | Cervical hyperaesthesia | MRI: Brain and cervical spinal cord showed caudal malformation of occipital bone ( Chiari-like malformation), herniation and compression of the cerebellum on the brainstem. MRI cervical segments and spinal cord showed also syringomylia | Pure, Pandy's test NA, no pleocytosis | no | CM/SM |
| 67D | Tremor, myoclonus, problems with ears | MRI: Brain and cervical segments of spinal cord showed caudal malformation of occipital bone (Chiari-like malformation), herniation of cerebellum and compression on brainstem. Cervical area of spinal cord showed dilatation of canalis centralis and signs of pre-syrinx | Pure, Pandy's test NA, no pleocytosis | no | CM/SM |
| 125D | Ataxia, motor deficits, fatigue, craniofacial deformity | MRI: Brain atrophy. Chiari-like malformation, cerebellar herniation. Spine and spinal cord normal | Pure, Pandy's test NA, no pleocytosis | no | CM |
| 138D | Ataxia, normoreflexia, motor deficits | MRI: Brain and cervical spinal cord showed caudal malformation of the occipital bone (Chiari-like malformation), dislocated cerebellum with compresion on the brainstem. MRI of thoraco-lumbar segments showed multiple degeration of intervertebral discs without compression of the spinal cord | Pure, Pandy's test NA, no pleocytosis | no | CM |
| 24D | Paraplegia, motor deficits, thoraco-lumbal hyperaesthesia | MRI: Thoracal, thoraco-lumbar, lumbar and lumbo-sacral segments and spinal cord showed multiple degeneration of intervertebral discs without compression of the spinal cord. Diffuse T2 hyperintense intramedullar lesion in the spinal cord | Pure, Pandy's test NA, mononuclear pleocytosis 41 cells/µL | no | M |
| 62D | Seizures, hypotrichosis | MRI: Normal | Pure, Pandy's test NA, no pleocytosis | yes | Controls |
| 21D | Seizures | MRI: Normal | Pure, Pandy's test NA, no pleocytosis | yes | Controls |
| 33D | Seizure, without other abnormalities | MRI: Normal | Pure, Pandy's test NA, no pleocytosis | yes | Controls |
| 38D | Seizures | MRI: Normal | Pure, Pandy's test NA, no pleocytosis | yes | Controls |
| 46D | Seizures, mild motor deficits | MRI: Normal | Pure, Pandy's test NA, no pleocytosis | yes | Controls |
| 53D | Seizure, fatigue | MRI: Normal | Pure, Pandy's test NA, no pleocytosis | yes | Controls |
| 55D | Seizure, fatigue, incontinence | MRI: Normal | Pure, Pandy's test NA, no pleocytosis | yes | Controls |
| 45D | Seizures | MRI: Normal | Pure, Pandy's test NA, no pleocytosis | yes | Controls |
| 47D | Seizures | MRI: Normal | Pure, Pandy's test NA, no pleocytosis | yes | Controls |
| 70D | Seizures | MRI: Normal | Pure, Pandy's test NA, no pleocytosis | yes | Controls |
| 92D | Seizures, limping on right hind limb | MRI: Normal | Pure, Pandy's test NA, no pleocytosis | yes | Controls |
| 93D | Seizures | MRI: Normal | Pure, Pandy's test NA, no pleocytosis | yes | Controls |
| 96D | Seizures | MRI: Normal | Pure, Pandy's test NA, no pleocytosis | yes | Controls |
| 99D | Seizures | MRI: Normal | Pure, Pandy's test NA, no pleocytosis | yes | Controls |
| 113D | Seizures | MRI: Normal | Pure, Pandy's test NA, no pleocytosis | yes | Controls |
| 124D | Seizures, oedema over right eye | MRI: Normal | Pure, Pandy's test NA, no pleocytosis | yes | Controls |
| 128D | Seizures | MRI: Normal | Pure, Pandy's test NA, no pleocytosis | yes | Controls |
| 129D | Seizures, bilateral cataracts, subcutaneus subject on the left side of the neck | MRI: Normal | Pure, Pandy's test NA, no pleocytosis | yes | Controls |
| 131D | Seizures, ataxia, motor deficits, limping, hyporeflexia | MRI: Brain normal, cervical segments with multiple degeneration of intervertebral discs without compression of the spinal cord | Pure, Pandy's test NA, no pleocytosis | yes | Controls |
| 140D | Seizures | MRI: Normal | Pure, Pandy's test NA, no pleocytosis | yes | Controls |
| 148D | Seizures | MRI: Normal | Pure, Pandy's test NA, no pleocytosis | yes | Controls |
| 154D | Seizures, bilateral cataracts | MRI: Normal | Pure, Pandy's test NA, no pleocytosis | yes | Controls |
| 162D | Seizures | MRI: Brain normal, ventriculomegalia (normal in Bulldogs) | Pure, Pandy's test NA, no pleocytosis | yes | Controls |
| 166D | Seizures, bilateral cataracts | MRI: Mild atrophy, otherwise normal | Pure, Pandy's test NA, normal, presence of activated macrophages | yes | Controls |
| 167D | Seizures, tremor, motor deficits, subsutaneus lump on ventral abdomen (1 cm diameter). | MRI: Normal | Pure, Pandy's test NA, no pleocytosis | yes | Controls |
| 172D | Seizures | MRI: Normal | Pure, Pandy's test NA, no pleocytosis | yes | Controls |
| 177D | Seizures | MRI: Normal | Pure, Pandy's test NA, no pleocytosis | yes | Controls |
| 181D | Seizures, tachypnoe, tachycardia | MRI: Normal | Pure, Pandy's test NA, mononuclear pleocytosis 6 cells/µL, presence of activated macrophages | yes | Controls |
| 183D | Seizures | MRI: Cortical atrophy, otherwise normal | Pure, Pandy's test NA, no pleocytosis | yes | Controls |
| 185D | Seizures | MRI: Normal | Pure, Pandy's test NA, no pleocytosis | yes | Controls |
| 189D | Seizures | MRI: Normal | Pure, Pandy's test NA, no pleocytosis | yes | Controls |
| 198D | Seizures | MRI: Normal, cervical segments and spinal cord showed dorsal compression of the spinal cord in the C1 region; this lesion was T2 hyperintense and T1 hypointense | Pure, Pandy's test NA, no pleocytosis | yes | Controls |
| 90D | Seizures, ataxia, cicling to the left, hiting subjects, hyperreflexia | MRI: Diffuse, massive, multiple T2 hyperintense and T1 hypointense lesions, which were saturated postcontrast | Pure, Pandy's test NA, mononuclear pleocytosis 150 cells/µL, presence of activated macrophages | yes | MUO |
| 19 | Behavioral normal, stand and walk normal,palpation without pain | MRI: Normal | Pure, Pandy's test negative, mononuclear pleocytosis 10 cells/µL | no | MUO |
| 22 | Motor deficits, paraparesis, vomitus | MRI: Degenerative changes on IVD without compresion on spinal cord, with detection T2 and STIR T2 hyperintense intramedullar lesion in L4-L5 | Pure, Pandy's test negative ,mononuclear pleocytosis 40 cells/3 µL | no | M |
| 30 | Hunchback poisture, motor deficits, vocalization | MRI: Hyperintense changes in rostral and dorsal cerebellum, cervical area normal | Pure, Pandy's test positive+, mononuclear pleocytosis 20 cells/µL | yes | MUO |
| 46 | Apathy, mild motor deficits , front limbs, palpation of Th-L area with discomfort | MRI: Thoraco-lumbar area with severe protrusion of intervertebral discs Th13-L1 and L1-2 with chronic compression of spinal cord, mild protrusion of L2-3 without compression of spinal cord | Pure, Pandy's test negative, no pleocytosis | no | M |
| 48 | Progresive motor deficits, excitation, disorientation, hyperactivity, circling to the left side | MRI: Intra-axial, T2 and FLAIR hyperintense lesions in rhinencephalon/lobus frontalis of left cerebral hemisphere, mild changes of left hippocampus | Pure, Pandy's test positive+, mononuclear pleocytosis 20 cells/µL | no | MUO |
| 50 | Mild disorientation, normal stance and gait, cataracts, central/peripheral blindness | MRI: Intra-axial, T2 hyperintense multifocal changes in white matter of cerebrum, dilation and asymmetrical changes of lateral ventricles (left bigger). Without neoplastis changes | Pure, Pandy's test positive+, mononuclear pleocytosis 10 cells/µL | no | MUO |
| 69 | Cervical pain, vocalisation and stiffnes , normal stance and gait | MRI: Cervical area with dissection of C6-C7 without protrusion /extrusion , spinal cord without compresion , but with mild T2 hyperintense intramedular diffuse changes in segments C3-C6, Cerebrum with multifocal T2 hyperintense changes -one with limited localisation, intra-axial lesion 10x8x7 mm without effect of mass in cranial area of left frontal lobus and without clear localisation , intra-axial T2 hyperintense diffuse lesion in thalamic area, close to 3. ventricle without mass effect . All intracranial lesions with mild hyperintensive signal on FLAIR sequence and hypointense on T1 sequence with postcontrast (Gadovist 1.2 ml i.v.) | Pure, Pandy's test positive+, mononuclear pleocytosis 40-60 cells/µL | no | MUO |
| 77 | Weak stance, excitation, mild disorientation, tremor, mild motor deficits | MRI: Cervical area withou abnormalities, intracranial multifocal, decent, without clear borders T2 hyperintense intra-axial lesions in cingular area , in right lobus piriformis on the level of Pons Varoli without dense contrast (Gadovist 2.3 ml i.v.) | Pure, Pandy's test positive+, mononuclear pleocytosis 300 cells/µL | no | MUO |
| 81 | Motor deficits, disrupted coordination | MRI: Multifocal T2 hyperintense changes in CNS of spinal segments Th10-11 , in cervical segments C3-4 and multifocal intracranial findings, filled right tympanic bulb | Pure, Pandy's test negative, mononuclear pleocytosis 10-20 cells/µL | no | MUO |
| 119 | Seizures, lung oedema, diarrhea | MRI: Normal | Pure, Pandy's test positive+, mononuclear pleocytosis 20 cells/µL, with presence of activated macrophages | yes | MUO |
| 83 | Neurologic deficits, mild paraparesis, circling to the side, sensory deficits | MRI: Lesion in brainstem without perilesional oedema, with not clear borders. Detection of T2 isointense, STIR hyperintense intramedullar lesions in L4-L5- L6. MRI of brain showed T2 and FLAIR hyperintesne, T1 isointense intra-axial lesion without dence contrast, parasagital left localisation in the brainstem. Neurocranium with detection of T2 hyperintense content in right tympanic bulb | Pure, Pandy's test negative, cytology with mild mononuclear pleocytosis | no | MUO |
| 106 | Seizures, normal stance and gait | MRI: Decent, multifocal, intra-axial, T2 hyperintense lesion without clear borders, without mass effect mainly in left hemisphere | Pure, Pandy's test negative, middle mononuclear pleocytosis 40 cells/µL | yes | MUO |
| 36 | Motor deficits of hind limbs | MRI: Multifocal T2 hyperintense intramedullar changes of Th-L segments of spinal cord, without detection any compression disease | Pure, Pandy's test positive+, mononuclear pleocytosis 30 cells/µL | no | M |
| 40 | Severe apathy, motor deficits, right anisocoria and fixed miosis | MRI: T2 mild diffuse hyperintense changes in periventricular space, in lobus piriformis and gyrus cinguli | Pure, Pandy's test positive+, severe mononuclear pleocytosis 50 cells/µL | no | MUO |
| 51 | NA | NA | NA | NA | MUO |
| 63 | Paraplegy, paraparesis, vomitus, diarrhea | MRI: Intramedullar T2 hyperintense changes in Th9-10 segments, severe anatomical abnormalities of thoracic and lumbar vertebrae and lumbo-sacral areas without compression of spinal cord | Pure, Pandy's test positive+ , mononuclear pleocytosis 20-30 cells/µL | no | M |
| 120 | Motor deficits, seizures, apathy, excitation, disorientation, circling and drifting to the right side | MRI: Brain with T2 and STIR hyperintense, diffuse changes present mostly in white matter of right hemisphere (leukoencephalopathy), without mass effect with heterogenic saturation postcontrast (Gadovist 0.33 ml i.v.) | Pure, Pandy's test very positive ++, mononuclear pleocytosis 50-100 cells/µL with prevalence of small and middle size lymphocytes, few activated macrophages | yes | MUO |
| 121 | Paraparesis, motor deficits | MRI: Thoraco-lumbar segments with multiple degeneration of intervertebral discs without compression of spinal cord | Pure, Pandy's test negative, mononuclear pleocytosis 20 cells/µL | no | M |
| 124 | Apathy, disorientation, vestibular ataxia, nystagmus | MRI: Brain structurally normal without abnormalities, stronger diffuse meningeal signal , T2 hyperintensive content of left tympanic bulb | Turbid, darker, Pandy's test very positive +++,massive lymphocytes pleocytosis 500-1000 cells/µL | no | MUO |
| 129 | Ataxia, motor deficits | MRI: Brain with mild cortical atrophy, expanded sulci of cerebral hemispheres, higher intensity of CSF in meningeal areas | Pure, Pandy's test very positive +++, massive mononuclear pleocytosis with prevalence of midle size lymphocytes 200 cells/µL | no | MUO |
| 139 | Seizures, circling, partial blindness, polymorbid patient, findings on mammary glands | MRI: Normal | Pure, Pandy's test negative, mononuklear pleocytosis 40 cells/µL, prevalence of lymphocytes and activated macrophages | yes | MUO |
| 140 | Seizures | MRI: Massive T2 and FLAIR hyperintense, symmetrical changes around falx cerebri in entire cingular cortex with decent saturation of focal contrast | Pure, Pandy's test negative, massive mononuclear pleocytosis 200 cells/µL | yes | MUO |
| 142 | Ataxia, motor deficits, seizure, circling to the left side | MRI: Brain with detection of multifocal T2 and FLAIR hyperintense, T1 isointense lesions in right and left hemispheres, in left Thalamus and brainstem. Massive dilatation and asymmetry of ventricular system in left half of the brain with atrophy. Susp. findings of necrosis | Pure, Pandy's test negative negative, cytology with detection of massive mononuclear pleocytosis cca 100 cells/µL, presence of big activated macrophages | yes | MUO |
| 149 | Motor deficits, cervical pain | NA | Pure, Pandy's test negative, no pleocytosis | no | MUO |
| 191 | Paraparesis, motor deficits, seizures, problem with defecation | MRI: Structural normal with meningeal saturation postcontrast (Gadovist 0.2 ml i.v.) | Pure, Pandy's test positive+, massive mononuclear pleocytosis 300-500 cells/µL with prevalence middle size lymphocytes | yes | MUO |
| 203 | Paraparesis, chronic motor deficits, frequent vomitus | MRI: Multiple degeneration of intevertabral discs with protrusion in segments Th9-10, L3-4 a L4- 5 with mild compression of spinal cord, T2 hyperintensive intramedular changes in Th9 segment | Pure, Pandy's test negative, moderate mononuclear pleocytosis 30-40 cells/µL | no | M |
| 220 | Symetric paraparesis, motor deficits | MRI: Multiple degeneration of intervertebral discs Th-L segments, disc without protrusion /extrusion , without myelopathy. Spinal cord in first lumbar segments with T2 hyperintense intramedullary changes with left localization | Pure, Pandy's test negative, moderate mononuclear pleocytosis 40 cells/µL with prevalence of small and middle size lymphocytes | no | M |
| 223 | Paraparesis, restlessness | MRI: Thoraco-lumbar segments with anatomical abnormalities - hemivertebrae Th8-Th10, degenerative changes on intervertebral discs with intramedullary T2 hyperintense changes | Pure, Pandy's test positive, moderate mononuclear pleocytosis 60 cells/µL | no | M |
| 231 | Apathy, motor deficits, circling, walking next to the wall | MRI: Brain with detection of multiple, not sharpe T2 and FLAIR hyperintense, T1 isointense lesion in right hemisphere, in right Thalamus massive lesion in brainstem without saturation in postcontrast. Lesion in right hemisphere with mild mass effect, perilesional oedema, compression of lateral ventricle and midline shift to the left | Pure, Pandy's test very positive +++, cytology with massive pleocytosis, cca 1500 cells/µL | no | MUO |
| 233 | Ataxia, seizures, tonic-clonic seizures, defecation | MRI: Multifocal changes in the brain , T2 hyperintense lesions of left frontal and temporal sulcus and right parietal sulcus with local malacia | Pure, Pandy's test negative, moderate mononuclear pleocytosis 20 cells/µL | yes | MUO |
| 239 | Motor deficits, painfull symetric spastic paraplegia | MRI: Thoraco -lumbar segments of spinal cord without comression myelopathy, from Th13 - L2 multifocal T2 hyperintensive intramedullar changes | Pure, Pandy's test negative, moderate mononuclear pleocytosis 40 cells/µL | no | M |
| 249 | Apathy, disorientation, motor deficits | MRI: Brain without structural abnormalities, T2 hyperintensive, intra-axial, decent lesion with unclear borders in diencephalon and pons Varoli, spinal cord in thoracolumbar and cervical segments with multiple degenerative changes of intervertebral discs without compression of spinal cord | Pure, Pandy's test positive+, mononuclear pleocytosis a 10-20 cells/µL | no | MUO |
| 80 | Tetraparesis, motor deficits, no consumption of food and drink | MRI: Normal | Pure, Pandy's test negative, mild mononuclear pleocytosis 20 cells/µL with activated macrophages | no | MUO |
| 98 | Spastic paraparesis, hypertermic, excitation | MRI: Multiple degeneration of intervertebral discs of Th segments without protrusion/extrusion , diffuse T2 hyperintense intramedullary changes | Pinkish with turbidness, Pandy's test very positive ++, robust mononuclear pleocytosis 100 cells/µL | no | M |
| 11 | Ataxia, mild impairment in coordination | NA | Pure, Pandy's test positive, mononuclear pleocytosis 20 cells/µL | no | MUO |
| 32 | Ataxia, motor deficits, cachexia, thoraco-lumbar kyphosis | MRI: Intra-axial, circular, well bounded, T2 and FLAIR hyperintense subject in left lateral ventricle , size 22x16x11 mm (lxwxh) with prominent mass effect, massive midline shift to the right without perilesional oedema and heterogenic saturation postcontrast (Gadovist 0.35 ml i.v.) | Pure, Pandy's test negative, mononuclear pleocytosis 30 cells/µL | no | Tumor |
| 60 | NA | NA | NA | NA | Tumor |
| 73 | Apathy, disorientation, pleurotonus | MRI: T2 and FLAIR hyperintense, T1 hypointense, intra-axial, not clear bounded circular lesion of right thalamus and lobus piriformis dexter, with massive mass effect with left midline shift and compression of right lateral ventricle with perilesional edema, size 2x2 cm, mild suggestion ring enhancement postcontrast (Gadovist 1.2 ml i.v.) | Pure, Pandy's test negative, mild reactive mononuclear pleocytosis 20 cells/µL with prevalence of activated macrophages | yes | Tumor |
| 75 | Apathy, disorientation, seizures, behavioral changes, right pleurotonus, blindness | MRI: T2 and FLAIR hyperintense, T1 hypointense, intra-axial circular mass in right lobus occipitalis with massive perilesional oedema,Thalamus and mesencephalon with massive mass effect with compression of right lateral ventricle and midline shift to the left swith ring enhancement postcontrast (Gadovist 2.2 ml i.v.) | Pure, Pandy's test negative, mild reactive pleocytosis 10 cells/µL | yes | Tumor |
| 97 | Seizures | MRI: Intra-axial, T2 and FLAIR hyperintense, T1 hypointense, bounded multilobular subject size 27x16x22 mm (hxwxl) on boarder of left lobus frontalis and rhinencephalon with massive perilesional oedema with mass effect in midline shift to the right and compression of the left lateral ventricle with diffuse saturation postcontrast (Gadovist 1.2 ml i.v.) | Pure, Pandy's test negative, no pleocytosis | yes | Tumor |
| 102 | Apathy, motor deficits, circling to the left | MRI: Massive, intra-axial, bounded oval mass in ventral area of frontotemporal part of the left hemisphere, size 27x25x27 mm (wxhxl), T2 and T1 isointense to the white matter, FLAIR hypointense, postcontrast (Gadovist 0.9 ml i.v.) | Pure, Pandy's test negative, reactive mononuclear pleocytosis 50 cells/µL | no | Tumor |
| 144 | Seizures, apathy | MRI: Detection of bounded, solitaire, circular, T2 and FLAIR hyperintense, T1 isointense lesion with perilesional oedema, without mass effect and detection of midline shift in the left lobus piriformis without saturation postcontrast | Pure, Pandy's test negative, no pleocytosis | yes | Tumor |
| 152 | Ataxia, excitation, nervous behaviour | MRI: Brain with detection suspected axial T2 and FLAIR hyperintense, T1 isointense circular bounded lesion with mass effect, midline shift to the right and with perilesional oedema on the border of brainstem and cerebellum area on the left side with massive saturation of the edge and cetral part of the lesion postcontrast, size 1.1x1.0x1.1 cm (wxhxl) | Pure, Pandy's test negative, no pleocytosis | no | Tumor |
| 159 | Seizures | MRI: T2 hyperintense, intra-axial, circular lesion with diameter 25 mm in right temporo-parietal area of the brain and diancephalon with massive mass effect with midline shift to the left and perilesional edema with transherniation with compression of right lateral ventricle, cranial segment of cerebellum and brainstem with mild ring enhancement postcontrast (Gadovist 6.5 ml i.v.) | Pure, Pandy's test negative, reactive pleocytosis | yes | Tumor |
| 192 | Hyperactivity in the night | MRI: Intra-axial, T2 and FLAIR hyperintense, circular subject with 10 mm diameter in the area of sella turcica under III. Ventricle | Pure, Pandy's test negative, no pleocytosis | no | Tumor |
| 198 | Ataxia, seizures, agressiveness | MRI: Intra-axial, T2 hyperintense, FLAIR heterogenic and T1 hypointense circular mass in right hemisphere, size 24x15x30 mm (hxwxl) with mass effect , midline shift to the left and massive perilesional edema without prominent saturation postcontrast | Pure, Pandy's test negative, no pleocytosis | yes | Tumor |
| 201 | Change in behavior | MRI: Intra-axial, bounded, circular subject in diencephalon in the position over sella turcica around III.ventricle , size 15x12x12 mm (wxhxl) on left side with mild mass effect and soft midline shift to the right without hydrocephalus, T2 hyperintense and isointensive centre, FLAIR hyperintense, T1 hypointense with isointense centre - with saturation postcontrast, hypophysis isolated | Pure, Pandy's test negative, no pleocytosis | no | Tumor |
| 204 | Apathy, disorientation, circling to the right | MRI: Intra-axial, well bounded circular subject with 2 cm diameter in the right hemisphere with massive mass effect and compression of right lateral ventricle and midline shift to the left, T2 and FLAIR hypointense, T1 isointense with saturation postcontrast (Gadovist 1.2 ml i.v.) with prominent ring enhacement, with massive peripheral oedema | Pure, Pandy's test positive+ , no pleocytosis | yes | Tumor |
| 208 | Apathy, mild motor deficits, spastic paraparesis | MRI: intra-axial, T2 hyperintense, unclearly bounded lesions in ventral position from III. Ventricle in the area of sella turcica, T1 isointense with diffuse saturation postcontrast (Gadovist 2.3 ml i.v.) | Pure, Pandy's test negative , no pleocytosis | no | Tumor |
| 210 | Seizures | NA | Pure, Pandy's test negative , no pleocytosis | yes | Controls |
| 219 | Seizures, pleurotonus to the left, right hemiparesis | MRI: Massive, intra-axial, T2 hyperintense lesion in range of whole left hemisphere with necrotic cavital centre with midline shift to the right and compression to the surrounding area | Pure, Pandy's test negative, moderate reactive pleocytosis 40 cells/µL | yes | Tumor |
| 225 | Apathy, ataxia, motor deficits, head tilt to the left, change in behavior | MRI: Intra/extra-axial, elipsoid, well bounded, T2/T1/FLAIR isointense (to the grey matter) subject with mass effect in the sella turcica, III. ventricle and diencephalon area, size 18 x 14 x 20 mm (wxhxl) with prominent perilesional oedema and diffuse saturation postcontrast with partly prominent ring enhancement (Gadovist 0.23 ml i.v.) | Pure, Pandy's test positive+, mild reactive pleocytosis 15 cells/µL | no | Tumor |
| 228 | Ataxia,pleurotonus to the left, impaired coordination, seizures | MRI: Intra/extra-axial, elipsoid, well bounded, T2/T1/FLAIR isointense (to the grey matter) subject with mass effect in the sella turcica, III. ventricle and diencephalon area, size 11 x 6 x 14 mm (wxhxl) with prominent perilesional oedema and diffuse saturation postcontrast with partly prominent ring enhancement (Gadovist 0.23 ml i.v.) | Pure, Pandy's test negative, mild reactive pleocytosis max 10 cells/µL | yes | Tumor |
| 237 | Ataxia, disorientation, behavioral change, motor deficits | MRI: Detection of solitary, oval T2 hyperintense, FLAIR hyperintense, T1 hypointense intra-axial lesion in the left hemisphere with prominent saturation postcontrast, FLAIR and T1 central hypointense signal with probable malacia. Lesion with prominent mass effect, compression of lateral venricle and perilesional oedema, size 1.2x1x2 cm (wxhxl) | Pure, Pandy's test negative, very mild pleocytosis 10-20 cells/µL | no | Tumor |
| 65 | Motor deficits, pruritus | MRI: Massive syrinx in segments C2-C4, 3 cm lenght, hight over 4 mm and wide over 5 mm, mild multiple degenetion of intervertebral discs, brain wih Chiari-malformation, obstructive hydrocephalus and mild and soft T2 hyperintense content of left tympanic bulb | Pure, Pandy's test negative, no pleocytosis | no | CM/SM |
| 72 | Hyperestesis of cervical area, motor deficits | MRI: Segments C1-Th4 with detection of syringomyelia. The most prominent dilatation of canalis centralis is observed C2-C4 level, with a diameter of 4.3 mm, in this range hyperintense signal of spinal cord on STIR sequence. Intervertebral disc degeneration of C6-C7 without compression of spinal cord. Mild dilatation of ventricle system. Chiari like malformation grade I. | Pure, Pandy's test negative, cytology normal, no pleocytosis | no | CM/SM |
| 101 | Apathy, ataxia, motor deficits, seizures | MRI: Massive asymetric hydropcephalus, massive dilatation of lateral venricle, T2 hyperintense content of the left tympanic bulb and massive cervical syringomyelia | Pure, Pandy's test negative, no pleocytosis | yes | SM |
| 141 | Motor deficits | MRI: Cervical and thoracal syringomyelia, segment C1 -wide of canalis centralis 2.8 mm, segment C2 -wide of canalis centralis 3.13 mm, Th 1 segment dilatation of canalis centralis 4.6 mm, Th 10 3.13 mm. Chiari like malformation gr. I, dilatation of ventricle system, presence of quadrigeminal cyst and dilatation of IV. lateral ventricle | Pure, Pandy's test negative, no pleocytosis | no | CM/SM |
| 174 | Paraplegia, polypnoe, motor deficits, excitation | NA | Pure, Pandy's test NA, mononuclear pleocytosis 100 cells/µL with prevalence of lymphocytes. | no | M |
| 12 | Seizures, right pupilla with miosis | MRI: Normal | Pure, Pandy's test negative, no pleocytosis | yes | Controls |
| 17 | Seizures, ataxia, CRT normal | MRI: Brain with mild cortical atrophy, othervise without abnormalities. Cervical segments with multiple degeneration of intervertebral discs | Pure, Pandy's test negative, no pleocytosis | yes | Controls |
| 41 | Seizures, no other abnormalities | MRI: Brain normal. Cervical segments without any degeneration of intervertebral discs | Pure, Pandy's test negative, no pleocytosis | yes | Controls |
| 59 | Seizures, no other abnormalities | MRI: Normal | Pure, Pandy's test negative, no pleocytosis | yes | Controls |
| 61 | Seizures, no other abnormalities | MRI: Normal | Pure, Pandy's test negative, no pleocytosis | yes | Controls |
| 62 | Seizures, no other abnormalities | MRI: Normal | Pure, Pandy's test negative, no pleocytosis | yes | Controls |
| 67 | Seizures, no other abnormalities | MRI: Normal | Pure, Pandy's test negative, no pleocytosis | yes | Controls |
| 100 | Seizures, pleurotonus to the left, rotation of left bulbus ocularis | MRI: Brain with mild cortical atrophy, othervise normal | Pure, Pandy's test negative, no pleocytosis | yes | Controls |
| 108 | Seizures, no other abnormalities | MRI: Normal | Pure, Pandy's test negative, no pleocytosis | yes | Controls |
| 111 | NA | NA | NA | NA | Controls |
| 29 | NA | NA | NA | NA | Controls |
| 26 | Seizures, nervous, disorientation, strikes to the subjects, especially in the night | MRI: Brain normal, mild asymmetry of lateral ventricles. Mild disection of intervertabral discs in C6-C7 segments without any protrusions/extrusions. Mild degeneration in L7-S1 segments without compession of cauda equina nerves | Pure, Pandy's test negative, no pleocytosis | yes | Controls |
| 53 | Seizures, no other abnormalities | MRI: Normal | Pure, Pandy's test negative, no pleocytosis | yes | Controls |
| 58 | Severe apathy, disorientation, motoric deficits, somnolence | MRI: Brain with dilatation of venricle sytem (VBHR-ventricle to brain height ratio over 30 %), without any structural abnormalities | Pure, Pandy's test negative, no pleocytosis | yes | Controls |
| 86 | NA | NA | NA | NA | Controls |
| 126 | Seizures, no other abnormalities | MRI: Normal | Pure, Pandy's test negative, no pleocytosis | yes | Controls |
| 127 | Seizures, no other abnormalities | MRI: Normal | Pure, Pandy's test negative, no pleocytosis | yes | Controls |
| 128 | Seizures, no other abnormalities | MRI: Normal | Pure, Pandy's test negative, no pleocytosis | yes | Controls |
| 136 | Seizures, no other abnormalities | MRI: Normal | Pure, Pandy's test negative, no pleocytosis | yes | Controls |
| 150 | Seizures, motor deficits, fearful | MRI: Normal | Pure, Pandy's test negative, no pleocytosis | yes | Controls |
| 161 | Seizures, motor deficits | MRI: Brain normal. Mild hydrocephalus. Mild T2 hyperintense content in the right tympanic bulb | Pure, Pandy's test negative, no pleocytosis | yes | Controls |
| 165 | One general seizure observed, since then no problems, bloodwork without any abnormalities, regular behaviour | MRI: Normal | Pure, Pandy's test negative, no pleocytosis | yes | Controls |
| 167 | Seizures, no other abnormalities | MRI: Normal | Pure, Pandy's test negative, no pleocytosis | yes | Controls |
| 173 | Seizures, no other abnormalities | MRI: Normal | Pure, Pandy's test negative, no pleocytosis | yes | Controls |
| 184 | Seizures, no other abnormalities | MRI: Normal | Pure, Pandy's test negative, no pleocytosis | yes | Controls |
| 193 | Seizures, mild apathy, tremor | MRI: Normal | Pure, Pandy's test negative, no pleocytosis | yes | Controls |
| 197 | Seizures, paroxysmal episodes | MRI: Normal, airy tympanic bulb | Pure, Pandy's test negative, no pleocytosis | yes | Controls |
| 218 | Seizures, no other abnormalities | MRI: Normal | Pure, Pandy's test negative, no pleocytosis | yes | Controls |
| 227 | Seizures, motor deficits, fearful | MRI: Brain normal, without structural changes, mild asymmetry of lateral ventricles | Pure, Pandy's test negative, no pleocytosis | yes | Controls |
| 234 | Seizures, hyperactive | MRI: Normal | Pure, Pandy's test negative, no pleocytosis | yes | Controls |
| 250 | Seizures, no other abnormalities | MRI: Brain normal, mild asymmetry of lateral ventricles | Pure, Pandy's test negative, no pleocytosis | yes | Controls |
| 252 | Seizures, ataxia, subjects on mammillary glands | MRI: Brain normal, mild asymmetry of lateral ventricles. Mild disecction of intervertabral discs in C6-C7 segments without any protrusions/extrusions. Mild degeneration in L7-S1 segments without compession of cauda equina nerves | Pure, Pandy's test negative, no pleocytosis | yes | Controls |
| 56 | Seizures, no other abnormalities | MRI: Normal | Pure, Pandy's test negative, no pleocytosis | yes | Controls |

Pandy's test (protein level): Positive: + (0.5-1 g/L), ++ (1-3 g/L),+++ (>3 g/L).

NA - not available
